# Properties of Layered TMDC Superlattices for Electrodes in Li-Ion and Mg-Ion Batteries

**DOI:** 10.1021/acs.jpcc.3c05155

**Published:** 2024-01-29

**Authors:** Conor Jason Price, Edward Allery David Baker, Steven Paul Hepplestone

**Affiliations:** Department of Physics, University of Exeter, Stocker Road, Exeter EX4 4QL, U.K.

## Abstract

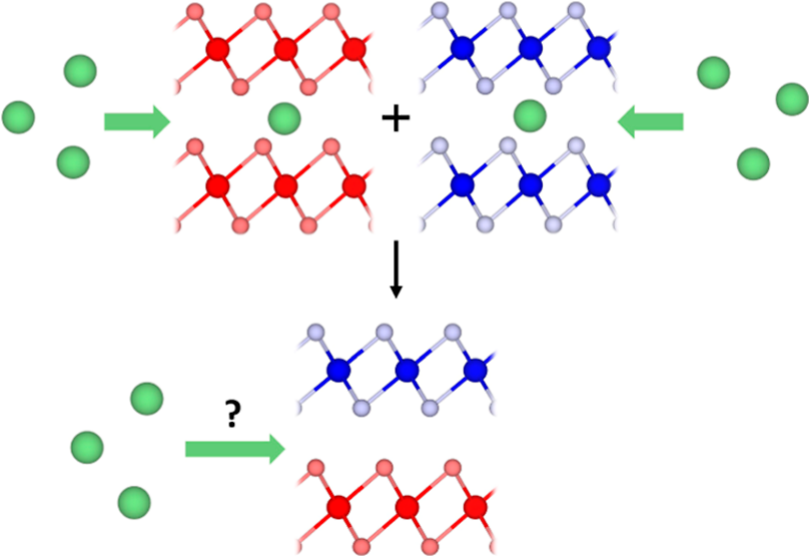

In this work, we present a first-principles investigation of the properties of superlattices made from transition metal dichalcogenides for use as electrodes in lithium-ion and magnesium-ion batteries. From a study of 50 pairings, we show that, in general, the volumetric expansion, intercalation voltages, and thermodynamic stability of vdW superlattice structures can be well approximated with the average value of the equivalent property for the component layers. We also found that the band gap can be reduced, improving the conductivity. Thus, we conclude that superlattice construction can be used to improve material properties through the tuning of intercalation voltages toward specific values and by increasing the stability of conversion-susceptible materials. For example, we demonstrate how pairing SnS_2_ with systems such as MoS_2_ can change it from a conversion to an intercalation material, thus opening it up for use in intercalation electrodes.

## Introduction

With the rise of renewable energy sources such as solar and wind power,^1,2^ and the growing popularity of electric vehicles,^3,4^ the need for cost-effective, efficient energy storage methods has increased dramatically. This has led to a substantial increase in battery research^5^ over the last two decades. Following the work of Whittingham^6,7^ and Goodenough^8,9^ in the 1970s and 1980s and the recent isolation of graphene by Novoselov and Geim,^10,11^ materials which possess a layered structure have received a lot of attention for energy storage. Materials such as NMC and its variants,^12−17^ the TMDCs,^18−20^ and the MXenes^21−24^ all demonstrate ideal electrode properties owing to the fact that their intrinsic structures possess van der Waals (vdW) gaps and provide natural channels for intercalated ions to occupy and travel through during the cycling of a cell. However, many of these materials possess voltages that lie outside ideal anode/cathode ranges, slow charging rates, and low capacities.

One clear extension to these layered materials can be achieved through the construction of superlattices and heterostructures. This allows for the utilization of not only the properties of the component materials but also the novel physics that can arise from their interface. The study of such nanocomposites has been facilitated by advances in fabrication techniques such as chemical vapor deposition,^25^ liquid exfoliation,^26^ and nucleation growth,^27^ which allow for monolayer control of material synthesis.^28−30^ The resultant electronic structure of vdW heterostructures is normally determined by Anderson’s rule^31−33^ with a few key considerations.^34−37^ The ability to predictably tailor the electronic properties has resulted in applications such as solar^38−43^ and photocatalytic^44−46^ cells, nanotransistors,^47−49^ and diodes.^50,51^

Heterostructures and superlattices have shown potential for electrode applications,^52−55^ with graphitic carbon being used as an additive to many MXenes^56−58^ and TMDCs such as MoS_2_^59,60^ and SnS_2_.^61−63^ Experimental investigations have shown improvements to the cyclability,^64^ and first-principles studies have found a reduction in volumetric expansions;^65,66^ hence, there is opportunity in using superlattices to enhance the performance of intercalation electrodes. However, due to the immense number of possible combinations, no study can be exhaustive, and previous works have been limited to a few select cases of heterostructures or superlattices. Nevertheless, a comprehensive study of TMDCs and their composites is needed to better understand how superlattice construction can enhance electrode properties.

Here, we report on a theoretical modeling of TMDC superlattices, with a focus on their properties for use as electrode materials in Li- and Mg-ion cells. We present the material voltage profiles, showing how these change between single constituents and compounds, and also discuss how the thermodynamic stability of these materials upon intercalation can be used as a way to estimate the charge storage capacity. We discuss other properties that are important for electrode materials, such as volumetric expansion and the resultant electronic structure, which is important for electronic conduction. This allows us to offer insight into how a careful choice of materials in a superlattice can be used to improve these materials for electrode applications.

## Methods

### First-Principles Methods

In this work, first-principles techniques based on density functional theory were used to determine the structural and energetic properties of superlattice structures composed of vertically stacked layered MX_2_ materials and to evaluate how these properties change when intercalated with varying levels of lithium or magnesium. We focus on superlattices with 1T-phase TMDC components as these are found to be the preferred phase in their pristine and intercalated forms, though it is worth noting that the Group VI TMDCs, lithium-intercalated Group V TMDCs, and magnesium-intercalated Group IV TMDCs will prefer the H-phase structure.^20^ While other phases^67,68^ are possible for the TMDCs, such as 2H and the α-NaFeO_2_-like structure, their intercalation environments are similar to those of the 1T phase, so only the 1T is considered. In this stacking configuration, the layers are arranged such that the metal atoms of each TMDC layer are vertically aligned, as in the usual T-phase structure.

**Figure 1:**
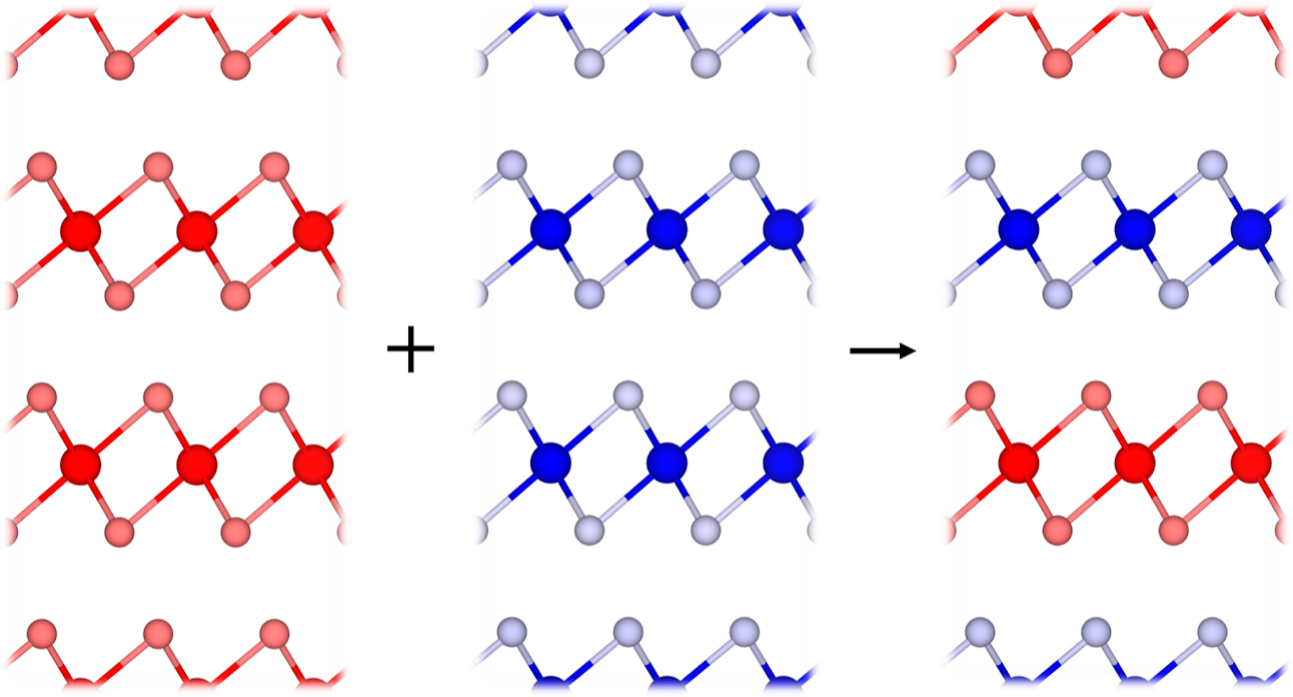
Schematic showing the 1:1 pairing of two lattice-matched TMDCs to form a superlattice. The layers are stacked such that the metal atoms of each layer are vertically aligned.

To investigate a range of superlattices based on TMDCs, we paired various TMDC materials with a second lattice-matched TMDC in a 1:1 match, as shown in Figure 1. To be considered “lattice-matched”, the two MX_2_-materials are required to have a lattice constant within 5% of each other. While the pairing of nonlattice-matched MX_2_-materials could be considered, there is a much larger combination space to investigate: phenomena such as moiré rotation effects,^69−71^ consideration of relative in-plane translations of the two atomic layers,^68^ and rippling^72,73^ are each deserving of a study of their own. Further to this, analysis of the effects of edge formation^74−76^ and the investigation of defects^77^ would also be required for a thorough description of a material in a working electrode. However, none of these can be explored until the fundamental properties of the core superlattices have been established. As such, we limit our investigation to pristine bulk superlattices formed through combinations of aligned MX_2_-materials with similar in-place lattice constants, with the addition of MoS_2_|SnS_2_, which shows strains between 5 and 10%. Further details of the material pairings, including the resultant strain and the formation energy, are presented in the Supporting Information.

To achieve a finer sampling of intercalant concentrations than would be accessible through consideration of only the primitive unit cells, supercells consisting of (2 × 2 × 2) unit cells were used for the individual TMDC materials, and supercells consisting of (2 × 2 × 1) unit cells were used for the superlattice structures. Each of these corresponds to 24 atoms, eight MX_2_ formula units, and two TMDC layers. These were then used as the bulk unit cells into which lithium and magnesium were intercalated for evaluation of voltages and thermodynamic stability, giving us access to a range of lithium concentrations between  MX_2_ and LiMX_2_ in increments of  MX_2_. Similarly, we can access magnesium concentrations between  MX_2_ and MgMX_2_ in increments of  MX_2_.

We determine the preferred sites of intercalation to be those with octahedral coordination, a further discussion of which is presented in the Supporting Information. Using the supercell sizes described above, we thus have access to eight potential octahedrally coordinated intercalation sites, which allow for 24 potential intercalant filling configurations. Each of these has been explored, and combinations of different concentrations have been used to emulate clustering effects.^78−81^ Further details of these are presented in the Supporting Information. For a given intercalant concentration, the configuration that results in the lowest energy structure is used for the evaluation of key electrode properties, such as calculation of the intercalation voltage and the assessment of the thermodynamic stability.

The calculations performed here employed the Vienna Ab initio Simulation Package (VASP).^82−85^ The valence electrons included for each species are indicated in the Supporting Information. The projector augmented wave method^86^ was used to describe the interaction between core and valence electrons, and a plane-wave basis set was used with an energy cutoff of 700 eV. All structural relaxations were completed using the Perdew–Burke–Ernzerhof (PBE)^87^ functional and converged to a force tolerance of 0.01 eV/Å per atom, while electronic self-consistency is considered to have an accuracy of 10^–7^ eV. Γ-centered Monkhorst–Pack grids^88^ of *k*-points equivalent to a 6 × 6 × 6 grid in the supercells are used throughout, and we have allowed for optimization of collinear spin. Van der Waals interactions have been addressed using the zero-damping DFT-D3 method of Grimme.^89^ Further calculation details are presented in the Supporting Information.

### Methods for Material Evaluation

To compare different levels of lithium-intercalated superlattice (SL = MX_2_M′X_2_^′^) the voltage, *V*, can be calculated using
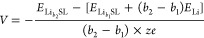
1for total lithium content *b*_2_ > *b*_1_, and energy of the superlattice structure with *b* intercalant atoms per SL formula unit . In this work, we consider values of 0 ≤ *b* ≤ 2, with *b* = 2 corresponding to one intercalant ion per metal atom of the host structure. *z* is the valency of the intercalant (*z* = 1 for the case of lithium, *z* = 2 for magnesium), and *E*_Li_ is the energy of a lithium atom as found in bulk. Each occurrence of Li should be replaced with an equivalent of Mg for magnesium intercalation. Further discussion of voltage calculation is presented in the Supporting Information.

Previous first-principles works^20^ have assessed the stability of individual TMDCs when intercalated with lithium or magnesium, with intercalation limits depending on how favorable the formation of secondary products (for example, Li_2_X or MgX) is. Typically, the formation of these compounds results in material amorphization and the irreversible loss of the layered TMDC structure. Here, we have developed a generalized approach for calculating *E*_IS_, a measure of stability and hence the reversible intercalation capacity of a material. This is given by
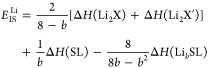
2for lithium intercalation and by
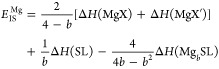
3for magnesium intercalation. In both of these, Δ*H*(A) gives the enthalpy of formation of compound A, *E*(A) gives the energy of compound A, and Δμ_B_ gives the chemical potential of elemental species B (in the intercalated SL structure) relative to its chemical potential when it is in its elemental bulk structure. *E*_IS_ is assessed for each intercalant concentration, with positive values indicating that the host TMDC is thermodynamically stable with intercalation and resists conversion to Li_2_S, MgS, or an equivalent compound. Negative values of *E*_IS_ then indicate that a material is susceptible to conversion, and the concentration at which *E*_IS_ is negative gives the limit of reversible intercalation. Further discussion of *E*_IS_ and its origins are provided in the Supporting Information.

## Results

### Volumetric Expansion

**Figure 2:**
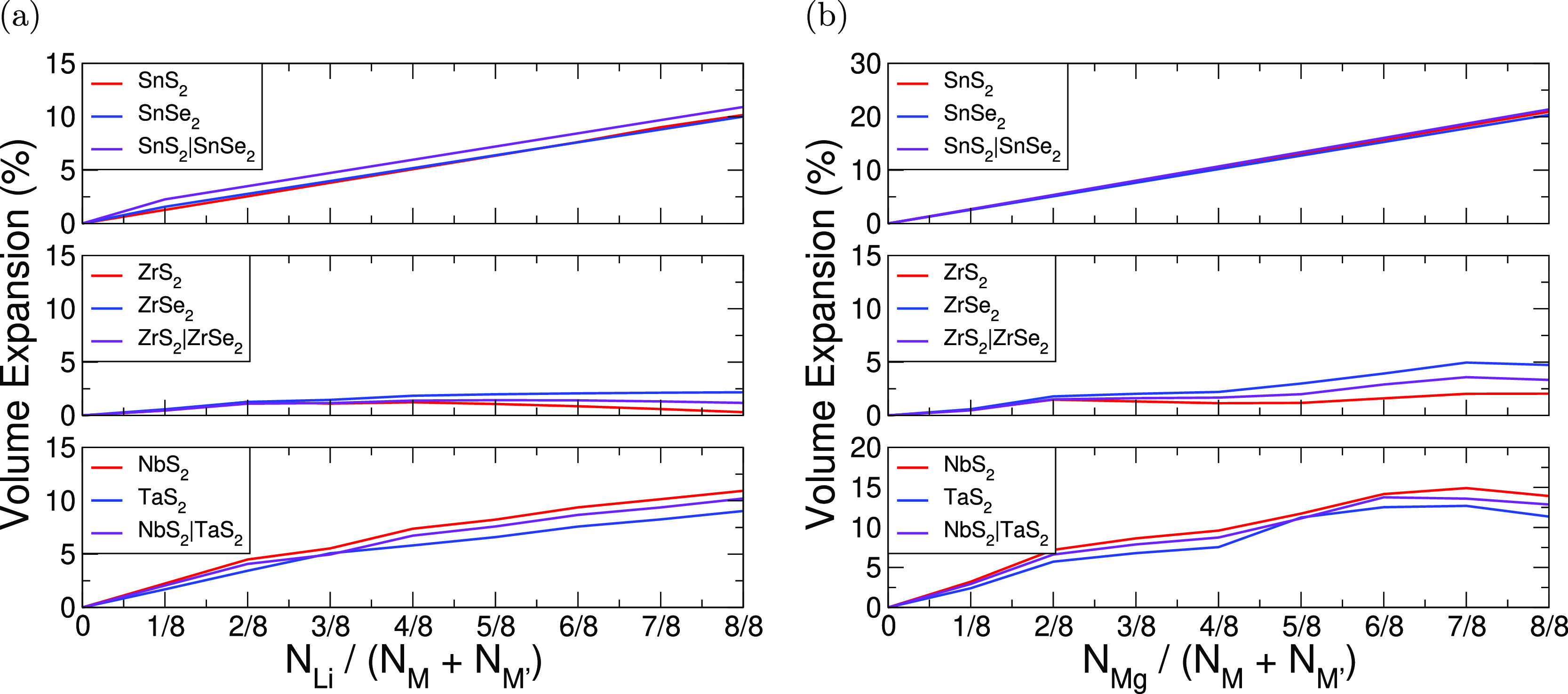
Volume expansion with intercalation for the selected TMDC superlattices, calculated with respect to the unintercalated structures using . (a) Data for lithium intercalation and (b) magnesium intercalation. In each of these, the *x*-axis gives the number of intercalant ions (*N*_Li/Mg_) per metal atom of the host structure (*N*_M_ + *N*_M′_).

One important metric for assessing the promise of a material for electrode applications is the volumetric expansion arising from intercalation. We calculate this expansion with respect to the unintercalated structure using  for initial volume *V*_0_ and the final volume *V*. Importantly, for electrode applications, we show that there is minimal volumetric expansion with the intercalation of these superlattices and highlight this with some examples in Figure 2. When intercalated with lithium (magnesium), we see that the SnS_2_|SnSe_2_ superlattice has a total volumetric expansion of 10.9% (21.4%), for ZrS_2_|ZrSe_2_, we see a total expansion of 1.8% (3.3%), and for NbS_2_|TaS_2_, we see a total expansion of 10.2% (12.9%). Thus, the minimal expansion demonstrated by layered materials holds upon construction of the superlattice, with most superlattices expanding by less than 20% (30%). These values are comparable with other layered materials that have demonstrated success as intercalation electrodes, including LiCoO_2_^90,91^ (2–3.25%), NMC^92^ (8.44%), and graphite^93^ (13.2%), as well as the <30% generally seen for the TMDCs.^20^

We note the surprising reduction in volume expansion of ZrS_2_ as lithium content increases beyond , and this behavior extends to the superlattice structure. The same can be seen as magnesium content increases beyond  (corresponding to the same amount of charge transfer to the host structure as  of lithium), though the volume increases again for larger intercalant concentrations. We also notice in Figure 2b a larger increase in the volume for SnS_2_ and TaS_2_ with magnesium intercalation beyond , and this behavior carries over to the corresponding superlattices.

A closer evaluation shows that for lithium intercalation, the volumetric expansions of the superlattices fall within 2% of the mean of the volumetric expansion for the relevant components. Thus, if the superlattice volumetric expansion were to be estimated by calculating the mean of the volumetric expansion arising in the component TMDCs, then we could expect the result to deviate by up to a 2% error from what is observed in the actual superlattice. This close agreement is not surprising considering the vdW gaps between MX_2_ layers.

We note a larger expansion upon magnesium intercalation than with lithium intercalation and attribute this to the greater charge donation from magnesium than with lithium. This can be seen in Figure 2. For example, SnS_2_|SnSe_2_ expands by 10.7% when half-intercalated with magnesium and 10.9% when fully intercalated with lithium (hence having similar levels of charge donation). Similarly, ZrS_2_|ZrSe_2_ expands by 1.7% when half-intercalated with magnesium and by 1.2% when fully intercalated with lithium, and NbS_2_|TaS_2_ expands by 8.7% when half-intercalated with magnesium and by 10.2% when fully intercalated with lithium. We rationalize this as the chalcogen species in the intercalated structures have larger negative charges, and the metal species have smaller positive charges. Consequently, there is a reduced attraction between the M and X species but an increased repulsion between the X species. This leads to a “stretching” of the MX_2_ layers along the *c*-axis. Comparable results are also seen for the other superlattices considered, further details of which are presented in the Supporting Information.

### Voltages

**Figure 3:**
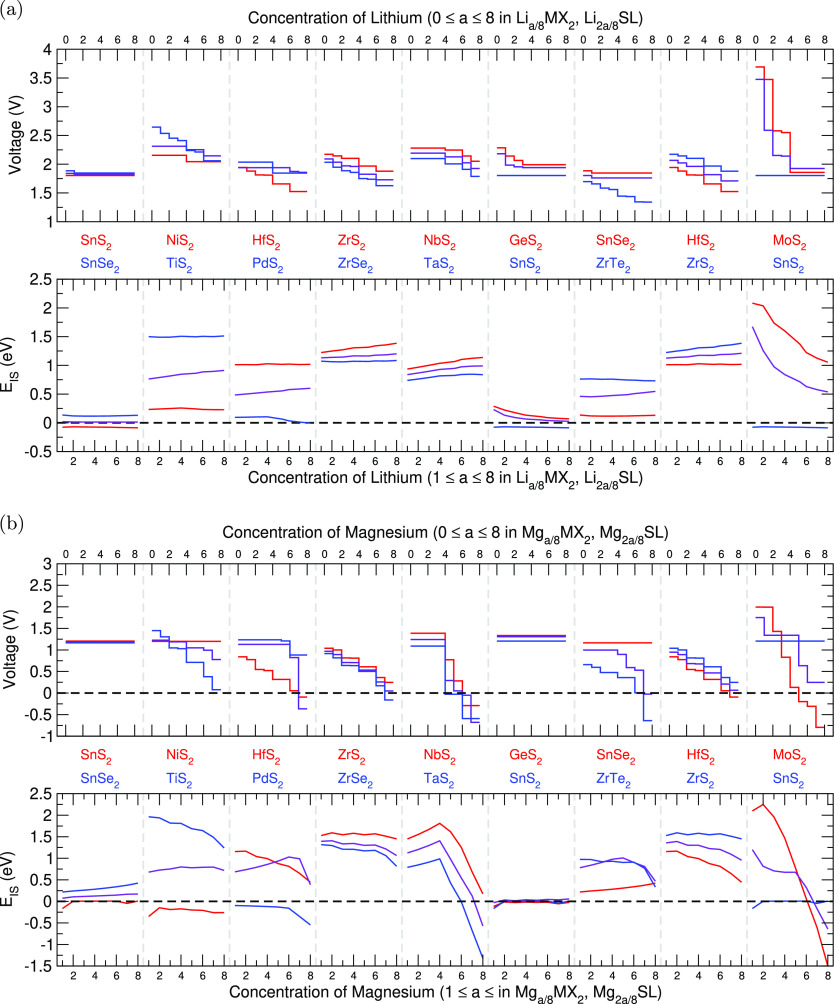
Intercalation voltages and *E*_IS_ for selected superlattices. (a) Results for lithium intercalation and (b) results for magnesium intercalation. In each of these, the top shows the voltage profile, and the bottom shows the variation of *E*_IS_ with intercalation. The superlattice data is presented in purple, and the data for the component materials is color-coded in red or blue.

In Figure 3, we present the voltage profiles and thermodynamic stability (indicated by *E*_IS_) of the highlighted superlattices with lithium and magnesium intercalation. For comparison, we have also included the results of the relevant individual TMDCs which were presented in our previous work.^20^ In general, we find that the intercalation voltage of the formed superlattice is intermediate with the profiles of the component materials. This is best highlighted with lithium-intercalated SnS_2_|SnSe_2_: SnS_2_ is shown to have a flat voltage profile at 1.80 V, and SnSe_2_ has a flat voltage of 1.85 V, with a minor step at the start of intercalation of 1.89 V. The superlattice then shows an almost flat voltage of 1.83 V, intermediate in value to those of the components in both shape and magnitude. We show a similar result for lithium-intercalated NbS_2_|TaS_2_, where the more distinct features of the NbS_2_ and TaS_2_ components are reproduced, maintaining the intermediate voltage profile.

We note that the voltage behavior seen with magnesium intercalation is very similar to that seen with lithium intercalation, and we highlight this with the SnS_2_|SnSe_2_ and GeS_2_|SnS_2_ structures. These show that the flat voltage profiles of the component materials result in a similarly flat voltage profile in the superlattices. Further, the dramatic drop in voltage for high magnesium concentrations in ZrTe_2_ produces a similar drop in voltage for high magnesium concentrations in the SnSe_2_|ZrTe_2_ superlattice. This SnSe_2_|ZrTe_2_ superlattice also suggests an exciting use of superlatticing: whereas the drop seen for ZrTe_2_ reaches a voltage of −0.64 V, the voltage drop demonstrated by the superlattice reaches a value of −0.02 V. Although this value is still negative, it suggests that the inclusion of SnSe_2_, a material that retains a constant voltage across the concentration range, limits the drop shown by the superlattice. The HfS_2_|ZrS_2_ further supports this: though the HfS_2_ component shows a negative voltage at high concentrations, the superlattice retains a positive value due to the inclusion of the ZrS_2_. This effect has been observed before, both using first-principles methods^94^ and experimentally.^64^

While some materials deviate from this with voltage profiles that extend beyond the bounds of the component materials, such as with the lithium-intercalated NiS_2_|TiS_2_ and MoS_2_|SnS_2_, and the magnesium-intercalated NbS_2_|TaS_2_ and MoS_2_|SnS_2_, these deviations do not remove the underlying shape of the component materials. Further, comparing the average voltages of the superlattice with those of the components highlights that taking an average of the component materials is a reliable method to predict the voltage of the formed superlattice. These comparisons, along with the results of 41 other superlattice structures which show the same result, are presented in the Supporting Information.

As mentioned above, electrode materials should ideally have a well-defined voltage,^95^ and so, based only on the voltage profiles in Figure 3a, pairings such as MoS_2_|SnS_2_ can be ruled out as a promising electrode material for lithium ion batteries. As one of the components (MoS_2_) has a large voltage variation, the resultant voltage for the superlattice can also be expected to have a large variation. Similarly, the magnesium-intercalation voltage profiles of NbS_2_|TaS_2_, SnSe_2_|ZrTe_2_, and MoS_2_|SnS_2_ vary significantly across the magnesium concentration range due to the large variation of one or both of the component TMDCs. However, this does also suggest that a large variation seen for a TMDC can be reduced by pairing with a TMDC with a constant voltage profile. For example, TiS_2_ varies by 0.59 V across the concentration range considered here. However, when paired with NiS_2_ (which varies by 0.11 V), the variation of the resultant NiS_2_|TiS_2_ superlattice is 0.17 V. Therefore, if a particular TMDC is desirable for use as an electrode but possesses a voltage which varies significantly, its voltage could be “pinned” by pairing it with a suitable partner.

The intercalation voltage of anode materials should be lower than 2 V, ideally in the range 0.5–1.5 V,^95^ and for cathode materials, it should exceed 3 V.^96^ As the pairing of two TMDCs results in a voltage that is intermediate to both, it would be sensible to combine materials that are energetically alike. For anodes, two TMDCs with low voltages should be combined, and for cathodes, two TMDCs with high voltages should be combined. If a low-voltage TMDC (e.g., SnS_2_ with a voltage of 1.80 V) were to be combined with a high-voltage TMDC (e.g., ScS_2_ with a voltage of 3.66 V^97^), the voltage of the superlattice (e.g., 2.69 V, see Supporting Information) would be poor for both anode and cathode applications.

### Thermodynamic Stability

The values of *E*_IS_ for a range of intercalant concentrations within the superlattices are shown in Figure 3a,b. As was demonstrated with the evolution of the intercalation voltage with concentration, the evolution of *E*_IS_ with intercalant concentration follows a trend that is an intermediate of the two component materials, and the value of *E*_IS_ of a superlattice at a given concentration is well approximated by calculating the average of the component materials. This suggests that, as *E*_IS_ is an indicator of the thermodynamic stability of a given TMDC against conversion, a highly stable material (characterized by a high, positive value of *E*_IS_) can be paired with a material that is susceptible to conversion (characterized by a low or negative value of *E*_IS_) to make a superlattice that is also resistant to conversion. This is shown with the pairing of SnS_2_|SnSe_2_, GeS_2_|SnS_2_, and MoS_2_|SnS_2_ with lithium intercalation. In each of these, SnS_2_ is the component with a negative value of *E*_IS_ across the range of lithium concentration. However, the formed superlattices have positive values of *E*_IS_, indicating the stability that has arisen from the inclusion of a thermodynamically stable component. We see the same result of a conversion-resistant component stabilizing a conversion-susceptible component with the pairings NiS_2_|TiS_2_ and HfS_2_|PdS_2_ for magnesium intercalation.

**Figure 4:**
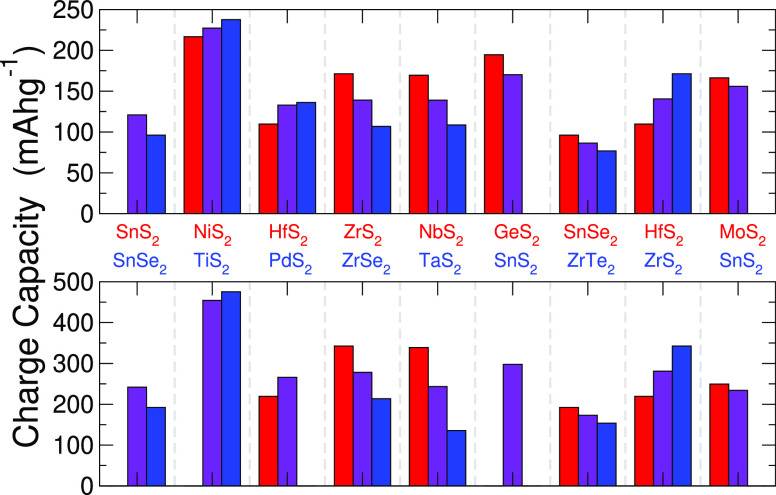
Reversible gravimetric charge capacity of selected superlattices and their component TMDCs for lithium (top) and magnesium (bottom) intercalation. The superlattice results are presented in purple, and the corresponding results for the component materials are presented in red and blue. Missing bars indicate materials with zero reversible capacity.

The importance of the improved resistance to conversion is highlighted by consideration of the gravimetric charge capacity, a quantity that is crucial for characterizing a material for electrode applications. We have used the range over which *E*_IS_ has a positive value to calculate the reversible gravimetric charge capacities for each of the superlattice structures, which are presented in Figure 4, along with the capacity of the component materials for comparison.^20^ Aside from the improvements in stability, we can also expect improvements in capacity simply due to the inclusion of a lighter material. For example, hafnium is a Period-VI element, so the specific capacity of 109.7 mA h g^–1^ for lithium intercalation (219.4 mA h g^–1^ for magnesium intercalation) is relatively low despite it possessing positive values of *E*_IS_ across the intercalation range. However, combining this with a TMDC composed of a lighter transition metal, such as ZrS_2_ in HfS_2_|ZrS_2_, increases this to 140.5 mA h g^–1^ (281.0 mA h g^–1^). Further, superlattice construction can, in some cases, provide a reversible charge capacity that is better than either of the components. This is highlighted by the intercalation of SnS_2_|SnSe_2_: SnS_2_ is shown to be susceptible to conversion reactions, so the capacity is zero. However, a combination with SnSe_2_ (which possesses a positive *E*_IS_ for both intercalants) results in a capacity of 120.9 mA h g^–1^ (241.8 mA h g^–1^). We can therefore improve not just the voltage (through “pinning”) and thermodynamic stability (by increasing *E*_IS_) of a TMDC through superlatticing but also the gravimetric charge capacity.

### Electronic Structure

One of the main reasons the TMDCs have received a lot of attention in recent years is for the wide range of electronic properties the family can exhibit, and their superlattices/heterostructures have been of further interest for the electronic physics that can arise from the combination of two materials.^34−37^ For electrode applications, electronically conductive materials are preferred so that compensating electrons from an external circuit can balance the positively charged lithium/magnesium ions.

We find that the electronic structure of a superlattice can be obtained crudely by superimposing the electronic structures of the constituent TMDC materials. As a result, combining TMDCs that offer a relative type II band alignment (staggered gap) results in a superlattice with a band gap that is smaller than either of the components, and combining a metallic TMDC with a TMDC that possesses a band gap results in a superlattice that is also metallic. Though exact band gap values can be sensitive to the choice of functional and the level of strain induced from lattice matching, this observation agrees with many previous works^36^ and shows that the construction of a superlattice provides a simple method through which the electrical conductivity can be improved.

**Figure 5:**
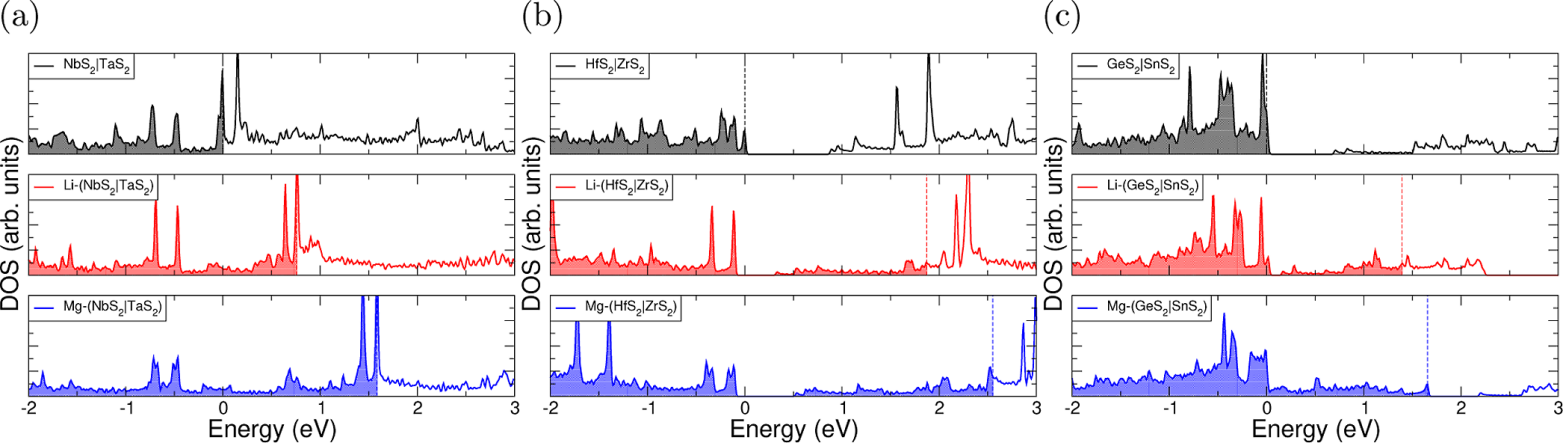
Electronic density of states (DOS) for pristine and intercalated superlattice structures. NbS_2_|TaS_2_ data is presented in (a), HfS_2_|ZrS_2_ in (b), and GeS_2_|SnS_2_ in (c). Pristine data are presented in black, data for lithium-intercalated structures in red, and data for magnesium-intercalated structures in blue. Each has been aligned with high-energy-occupied states of the pristine superlattice material. The energy of the highest occupied state (*E*_HOMO_) is indicated with dashed lines. Corresponding band structures are presented in the Supporting Information.

The introduction of ionic species into the host structure dramatically changes the nature of interlayer bonding, however, and consequent changes to the electronic structure can be expected. Here, we investigate how the electronic structure of superlattice structures changes with intercalation. In Figure 5, we present the electronic structure density of states (DOS) for NbS_2_|TaS_2_ (5a), HfS_2_|ZrS_2_ (5b), and GeS_2_|SnS_2_ (5c). These show the electronic DOS for the intercalated superlattices, along with the limit of lithium and magnesium intercalation corresponding to one intercalant per metal atom in the host supercell. The associated electronic band structures are presented in the Supporting Information. We have qualitatively aligned to the high-energy occupied states of the unintercalated superlattice at Γ, allowing us to comment on the relative position of the highest occupied molecular orbital (HOMO) level, with further details presented in the Supporting Information. We emphasize that this is an aesthetic choice made purely for easier comparison of the highest-occupied states of the pristine and intercalated materials.

We identify several broad groups describing the electronic behavior: the superlattice either (i) retains a conductive nature with intercalation, (ii) undergoes a semiconductor to conductor transition, (iii) possesses an insulating nature before intercalation and at the intercalation level corresponding to one intercalant per metal atom in the host supercell, or (iv) undergoes a conductor to semiconductor transition. The superlattice NbS_2_|TaS_2_ is an example of group (i), possessing no band gap at the start and end of intercalation. We can see from Figure 5a (and the corresponding band structure in the Supporting Information) that this is due to the HOMO lying in the middle of a linear band that extends from −0.5 eV at M to 2.5 eV between K and Γ. Electrons that are transferred from the intercalants to the host then simply occupy the unoccupied states in this band, so the HOMO level progressively rises. For HfS_2_|ZrS_2_, an example of group (ii), we see a very similar behavior, but the presence of an initial band gap means that there is a much larger initial jump in the position of the HOMO. However, the continuous range of bands beyond this allows for a gradual rise in the HOMO level, as seen for group (i). This is presented in Figure 5b. Figure 5c shows the electronic DOS for the GeS_2_|SnS_2_ superlattice and its intercalated forms, where we see the pristine structure possesses a band gap of ∼0.6 eV. Upon lithium intercalation, the HOMO shifts to intersect the two lowest-energy unoccupied states of the pristine structure, becoming metallic as with a type (ii) material. However, upon intercalation with magnesium, these states become fully occupied, and the HOMO level then sits at the bottom of a further band gap.

Though HSE and GW calculations typically offer improvements in accuracy over LDA and GGA functionals, this is not universally true for the TMDCs. Some works have found cases of GGA functionals producing band gaps closer to experimental values than HSE^98^ or GW^99,100^ calculations. Our previous work^36^ considered the band alignment of TMDCs and also found that HSE calculations resulted in the same conclusions as those obtained in the PBE functional. As such, we do not use these higher levels of calculation as there is not a guaranteed improvement in the results, nor do we expect any changes to the conclusions already presented.

### Further Considerations

Beyond the above discussions of volumetric expansion, intercalation voltage, stability, and electronic structure, all of which are important considerations for any electrode material, we here present a brief discussion of diffusion characteristics, elastic properties, and charge transfer, as these can also play an important role in electrode function. Further details of these are presented in the Supporting Information.

Climbing-image nudged elastic band^101^ calculations were used to identify the octahedrally coordinated intercalation site to be the lowest in energy, with ionic diffusion predominantly occurring between adjacent octahedral and tetrahedral sites. The barriers to this diffusion in the superlattices are shown to be intermediate with their TMDC components. Thus, due to the exponential dependence on the diffusion barrier in the Arrhenius rate, the rate of diffusion through a superlattice is lower than the average of the rates of the two components but faster than the rate of the component with the largest barrier.

We also calculate the elastic tensor of the superlattices discussed above. We find all of these superlattices to be elastically stable, with the exception of magnesium-intercalated HfS_2_|PdS_2_ and magnesium-intercalated MoS_2_|SnS_2_. The bulk, shear, and Young’s moduli generally increase with the addition of an intercalant, with larger values obtained with magnesium intercalation, indicating the strengthened interaction between TMDC layers due to the addition of an ionic intercalant. The elastic values of the superlattices are again found to be intermediate to those of the component TMDCs.^102^

Finally, we also present charge analysis in the Supporting Information as this can offer insight into the electronic structure and intercalation properties. As there is relatively little charge transfer between component layers upon superlattice construction, there is correspondingly minimal change to the electronic structure of component TMDCs upon construction of a superlattice/heterostructure.^36,37^ Consequently, the evolution of the superlattice electronic structure with intercalation is very similar to what has been observed for the component materials,^20^ i.e., the donated electrons from intercalated lithium or magnesium gradually fill the unoccupied states of the host structure, as discussed above. We find that the charge transfer from the intercalant to the host material is intermediate to the charge transfer of the component layers, which explains the observed “averaging” of the electrode properties of volumetric expansion, voltage, and stability assessed here.

## Conclusions

We have presented here the results of an investigation of intercalation into transition metal dichalcogenide superlattices with both lithium and magnesium. The volumetric expansion, electronic structure, intercalation voltages, and thermodynamic stability determined through phase diagrams have all been considered, as the information they provide is essential for the consideration of materials for use as electrodes. Upon construction of a superlattice, we find that many of these properties can be well approximated through the consideration of the equivalent properties for the component layers. For example, if the superlattice volumetric expansion were to be estimated by calculating the mean of the volumetric expansion arising in the component TMDCs, we could expect the result to deviate by up to a 2% error from what is observed in the actual superlattice, and the voltage profiles of the component materials provide bounds to the voltage profile exhibited by the constructed superlattice. Further, the unoccupied states of the host material are progressively filled with the addition of an intercalant, which follows the behavior observed with the individual TMDCs. Most interestingly, the construction of superlattices allows for many improvements to component materials: the construction of a superlattice can result in a reduction of the electronic band gap, thus improving electronic conductivity; conversion-resistant materials can be used to increase the stability of conversion-susceptible materials, extending their cyclability and lifetime; and materials can be chosen such that the overall voltage can be tuned toward specific values.

The conclusions presented in this work should also extend to other layered materials. In particular, the layered transition metal oxides offer a group of materials that are very closely related to the TMDCs used to construct the superlattices here and have already demonstrated success as electrodes. Using the ideas used here, however, they could have their voltages tuned, their intercalation stability improved, and ultimately have an increased energy storage capacity.

## Supporting Information Available

Further details of the methodology, including the results of nudged elastic band calculations and the derivation of limits for thermodynamic phase diagrams, calculation of strain and formation energy associated with constructing each superlattice, numerical values of the results presented in the main article, along with calculated elastic properties and charge analysis, and relevant electrode properties of an additional 41 superlattice structures (PDF)
