# Supplementary material for: Properties of Layered TMDC Superlattices for Electrodes in Li-Ion and Mg-Ion Batteries
Source: J Phys Chem C Nanomater Interfaces. 2024 Jan 29;128(5):1867–76. doi: 10.1021/acs.jpcc.3c05155 (PMC10860140; doi:10.1021/acs.jpcc.3c05155)
Supplement: Supplementary file 1 — jp3c05155_si_001.pdf [file jp3c05155_si_001.pdf]

Supporting Information for: "Properties of Layered TMDC Superlattices for Electrodes in Li-Ion and Mg-Ion Batteries"

Conor Jason Price,* Edward Allery David Baker, and Steven Paul Hepplestone*

Department of Physics, University of Exeter, Stocker Road, Exeter, EX4 4QL, United Kingdom

E-mail: cjp225@exeter.ac.uk; S.P.Hepplestone@exeter.ac.uk

Methodology

Calculation Details

The calculations performed here employed the Vienna Ab initio Simulation Package (VASP).[1–4] The valence electrons considered in the DFT calculations for each species considered in this work are presented in Table S1. The projector augmented wave method[5] was used to describe the interaction between core and valence electrons, and a plane-wave basis set was used with an energy cutoff of 700 eV. All structural relaxations were completed using the Perdew-Burke-Ernzerhof (PBE)[6] functional, and converged to a force tolerance of 0.01 eV/Å per atom, while electronic self-consistency is considered to an accuracy of 10^{-7} eV. Monkhorst-Pack grids[7] of k-points equivalent to a $6 \times 6 \times 6$ grid in the supercells are used throughout, and we have allowed for optimisation of collinear spin. Van der Waals interactions have been addressed using the zero damping DFT-D3 method of Grimme.[8] All structural relaxations were completed using the PBE functional[6] using the conjugate gradient algorithm and converged to a force tolerance of 0.01 eV/Å per atom, and electronic self-consistency is considered to an accuracy of 10^{-7} eV. For calculation of the elastic tensor, the primitive unit cell structures were geometrically relaxed to a stricter force convergence of 0.0001 eV/Å per atom, using Monkhorst-Pack k-point grids of $12 \times 12 \times 6$. The PBE functional and DFT-D3 method of Grimme were again used.

Typically, alignment of the electronic structures of different materials would be achieved either through alignment of core states[9] or with respect to the vacuum level.[9–11] Such approaches have been successful in comparing surfaces with bulk materials, and comparing mono/bilayer TMDCs with formed heterostructures. However, as we show later, the charge donation arising from intercalation makes quantitative alignment of the pristine and intercalated structures extremely difficult. In Figure S15 we see the introduction of an intercalant results in regions of electron accumulation and depletion. This occurs most dramatically around the chalcogen species, as highlighted with the red and blue regions in Figure S15c

and Figure S15d. The electric field arising from this redistribution of charge will shift the states associated with the chalcogen atoms. However, as the redistribution of charge around the metal species differs from that around the chalcogen, the states associated with the metal will be shifted to a different extent. Alternatively, we could align with respect so some absolute reference level, such as the vacuum level. Unfortunately, there is no way to construct a slab/surface system for these intercalated structure without inducing a significant electric field across the vacuum region (caused by positively-charged intercalant ions on one surface and negatively-charged chalcogen ions on the opposing surface) or by breaking stoichiometry (by constructing mirror-image surfaces).

As the charge distribution and the consequent electric fields in the systems considered in this work is significant (unlike those which arise with the combination of mono/bilayer TMDCs into a heterostructure), and it is not uniform across the system (as for comparing surfaces to bulk systems), we do not see the alignment of core states as an appropriate method for a quantitative comparison of the electronic structures.

Instead, we have qualitatively aligned to the high-energy occupied states of the unintercalated superlattice at Γ, allowing us to comment on the relative position of the highest occupied molecular orbital (HOMO) level. We emphasise that this is an aesthetic choice done purely for easier comparison of the highest occupied states of the pristine and intercalated materials.

Intercalation Sites

We focus on superlattices with 1T-phase TMDC components as we generally found these to be the preferred phase in their pristine and intercalated forms, though it is worth noting that the Group VI TMDCs, lithium-intercalated Group V TMDCs, and magnesium-intercalated Group IV TMDCs will preference the H-phase structure .[12] Supercells of $(2 \times 2 \times 1)$ superlattice unit cells (corresponding to 24 atoms, eight MX_2 formula units, and two TMDC layers) were generated and structurally relaxed. Previous investigations into the intercala-

Table S1: Electronic configurations of electrons modelled for different species considered in this study.

Species	Included Electrons	Species	Included Electrons
Li	$1s^2 2s^1$ (3)	Re	$5d^6 6s^1$ (7)
Mg	$2p^6 3s^2$ (8)	Fe	$3d^7 4s^1$ (8)
S	$3s^2 3p^4$ (6)	Ru	$4s^2 4p^6 4d^7 5s^1$ (16)
Se	$4s^2 4p^4$ (6)	Os	$5d^7 6s^1$ (8)
Te	$5s^2 5p^4$ (6)	Co	$3d^8 4s^1$ (9)
Sc	$3d^2 4s^1$ (3)	Rh	$4d^8 5s^1$ (9)
Y	$4s^2 4p^6 4d^2$ (10)	Ir	$5d^8 6s^1$ (9)
Ti	$3p^6 3d^3 4s^1$ (10)	Ni	$3d^9 4s^1$ (10)
Zr	$4s^2 4p^6 4d^3$ (11)	Pd	$4d^9 5s^1$ (10)
Hf	$5d^3 6s^1$ (4)	Pt	$5d^9 6s^1$ (10)
V	$3d^4 4s^1$ (5)	Cu	$3d^{10} 4s^1$ (11)
Nb	$4s^2 4p^6 4d^4 5s^1$ (13)	Ag	$4d^{10} 5s^1$ (11)
Ta	$5d^4 6s^1$ (5)	Au	$5d^{10} 6s^1$ (11)
Cr	$3s^2 4s^1 4p^6 4d^5$ (14)	Ge	$3d^{10} 4s^2 4p^2$ (14)
Mo	$4d^5 5s^1$ (6)	Sn	$4d^{10} 5s^2 5p^2$ (14)
W	$5d^5 6s^1$ (6)	Pb	$5d^{10} 6s^2 6p^2$ (14)
Mn	$3p^6 3d^6 4s^1$ (13)		

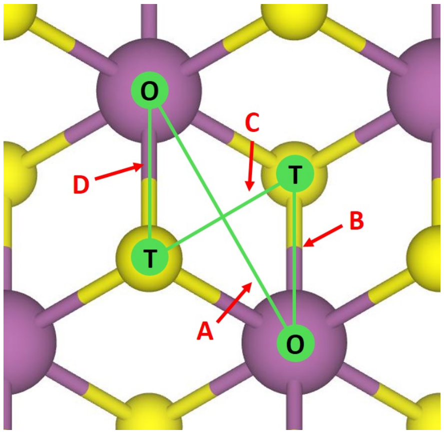

Figure S1: Different CI-NEB routes (A, B, C, D) considered, between different octahedral (denoted 'O') and tetrahedral (denoted 'T') sites.

tion of layered TMDCs have determined two different sites for intercalation, one where the intercalant has octahedral six-fold coordination with the chalcogen species of the host, and one with tetrahedral four-fold coordination. Of these, the octahedral site is widely observed to be preferred due to its higher coordination. We have performed a similar investigation into selected superlattices, employing climbing-image nudged elastic band (CI-NEB) calculations[13] along three different routes between two unique intercalation sites, see Figure S1. The key difference between CI-NEB and the standard NEB method is that the first stage of the CI-NEB is to find a maximal energy point along the reaction route. This prevents the elastic band/beads to shift to a lower energy pathway, giving a clearer description of maximal and saddle points. The available intercalation sites are the octahedrally coordinated site above the metal atom, labelled O, and the tetragonally coordinated site above the chalcogen atom, labelled T. The CI-NEB routes between two equivalent O-sites (Route A), between adjacent O- and T-sites (Route B), and between two equivalent T-sites (Route C) were considered for selected superlattices. Due to the asymmetry of adjacent layers, there is also another route between adjacent O- and T-sites that is distinct from Route B. We label this as Route D. These routes are depicted in Figure S1.

Using the results of the CI-NEB calculations, we can also comment on the ionic rates of diffusion through the superlattices. We first note from Figure S2 that, whilst the T-site is

higher in energy, the barrier between an O- and T-site is lower than the barrier between two O-sites. As the rate of diffusion is governed by the Arrhenius-type equation, Route B would be the dominant diffusion pathway due to its lower activation barrier. Generally, we note that the diffusion barrier for the superlattice is intermediate to the barriers arising from the component TMDCs. Using the example of the $SnS_2|SnSe_2$ superlattice shown in Figure S3, the barrier to lithium (magnesium) diffusion along Route A is 1.25 eV (2.34 eV), whereas it is 1.10 eV (2.20 eV) in SnS_2 and 1.34 eV (2.43 eV) in $SnSe_2$. Along Route B these values are 0.52 eV (1.00 eV) in the superlattice, 0.47 eV (1.00 eV) in SnS_2, and 0.59 eV (1.01 eV) in $SnSe_2$. Similarly along Route C these values are is 0.56 eV (1.03 eV) in the superlattice, 0.47 eV (1.00 eV) in SnS_2, and 0.59 eV (1.01 eV) in $SnSe_2$. In a single TMDC, diffusion routes B and D are equivalent, and hence the diffusion barriers are identical. Due to the chemical and structural similarity of SnS_2 and $SnSe_2$, routes B and D are very similar in Figure S2a. However, other pairings which involve more dissimilar TMDCs demonstrate a greater asymmetry between these routes, as shown in Figure S2. Due to the rate of diffusion following an Arrhenius equation, these asymmetries can lead to a significant bias to diffusion along different routes. Due to the exponential dependence on the diffusion barrier in the Arrhenius rate, the rate of diffusion through a superlattice (with the barrier being approximately the average of the two components) is lower than the average of the rates of the two components.

Using the above routes , we present the resultant barrier heights for selected superlattices in Figure S2. For both lithium and magnesium, these results show that the O-site is the most favourable site for intercalation due to a higher Li-S and Mg-S coordinations, and a larger volume for intercalation.[14] Hence the O-site has been used for the following study. The supercell size used provided eight potential (octahedrally-coordinated) intercalation sites, corresponding to the 24 potential filling configurations presented in Figure S4 and Table S2, which were all explored.

It has previously been observed that intercalants can cluster rather than homogeneously

Table S2: Table showing the 24 different intercalation configurations (plus the unintercalated) considered for the intercalation of MX_2 materials for nine different lithium concentrations.

No. Li Atoms	Sites Filled	No. Li Atoms	Sites Filled
0	-	4	adeh
1	a	4	adfg
2	ab	4	adfh
2	ae	5	abcde
2	af	5	abcef
2	ah	5	abceh
3	abc	6	abcdef
3	abe	6	abcefg
3	bce	6	abdefg
4	abcd	6	bcdefg
4	abce	7	abcdefg
4	abch	8	abcdefgh

distributing throughout a host structure,[12,15–18] and so taking the difference between two equivalent structures of consecutive intercalant contents does not always give the most accurate representation of what happens in reality. For example, it may be more favourable for an intercalant (e.g. Li) to fill one cell to $Li_{\frac{0}{8}}MX_2$ and an adjacent cell to $Li_{\frac{8}{8}}MX_2$, rather than filling a both cells to $Li_{\frac{4}{8}}MX_2$. This would indicate that the intercalant prefers to cluster or phase separate. This has therefore also been considered in the evaluation of electrode properties.

Across the different intercalant configurations and the allowing for clustering by considering combinations of different intercalant concentrations, the lowest energy arrangement of intercalant ions has been used for the assessment of electrode properties.

Details of Materials Used in Superlattices

To investigate a range of superlattices based on SnS_2, we paired SnS_2 with a second lattice-matched MX_2-material in a 1:1 match, as shown in Figure 1 of the main article. To be considered "lattice-matched", the second MX_2-material was required to have a lattice constant

within 5% of the lattice constant of SnS_2. The considered lattice-matched MX_2-materials (and their % mismatch with SnS_2) were HfS_2 (1.85%), ScS_2 (0.82%), $SnSe_2$ (4.83%), $TiSe_2$ (4.18%), $TiTe_2$ (1.74%), ZrS_2 (0.57%), and $ZrSe_2$ (2.51%). NiS_2 and MoS_2 lie outside of this restriction, with matches of 8.00% and 13.81% respectively, but have also been included. We model these superlattices as supercells of 2×2 $SnS_2|MX_2$ unit cells (24 atoms, eight MX_2 formula units, and two MX_2-material layers). These supercells were compared with supercells of the bulk constituents of the same size.

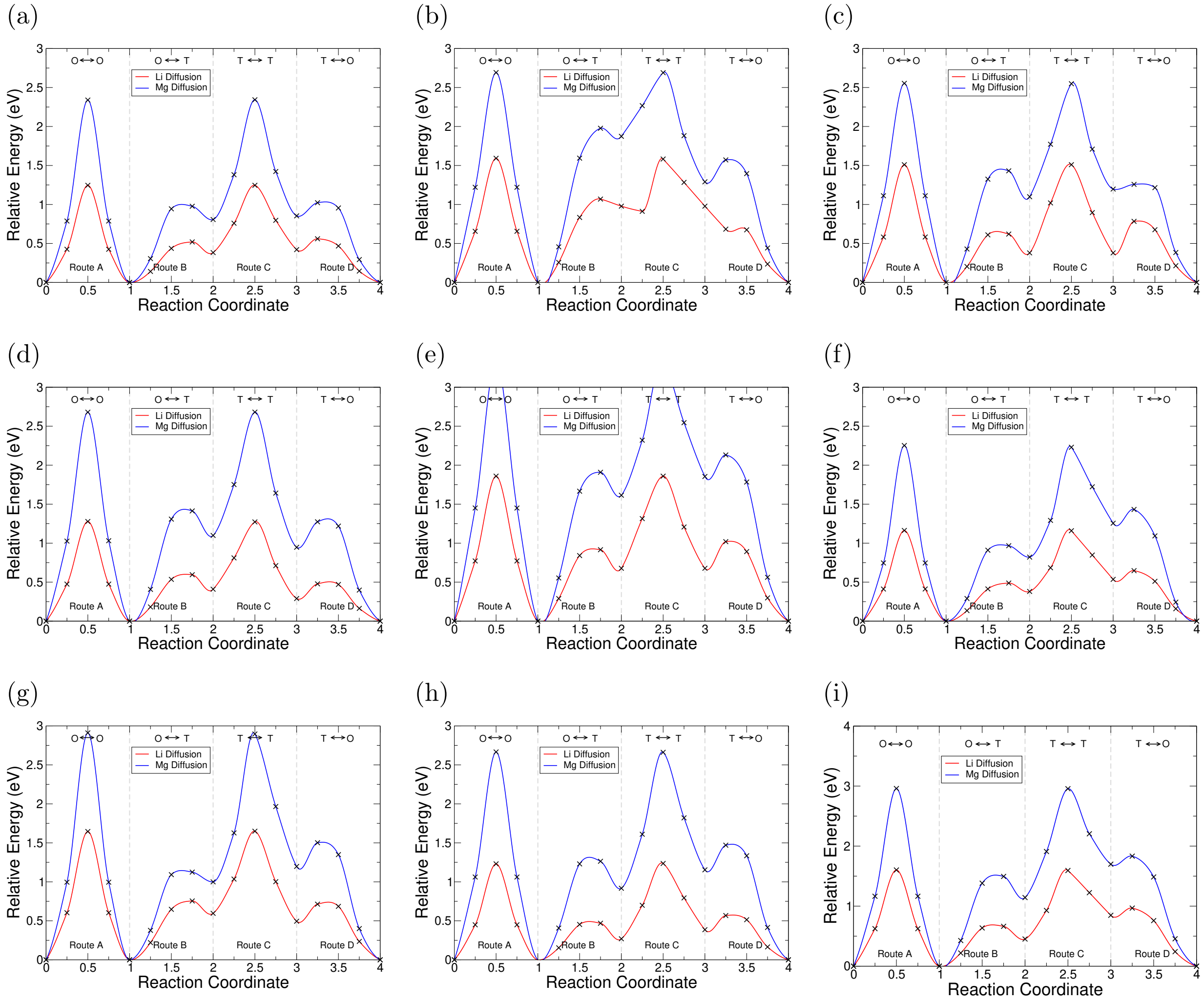

Figure S2: Diffusion barrier heights calculated using the CI-NEB method for lithium and magnesium in the highlighted superlattice materials, $SnS_2|SnSe_2$ (S2a), $NiS_2|TiS_2$ (S2b), $HfS_2|PdS_2$ (S2c), $ZrS_2|ZrSe_2$ (S2d), $NbS_2|TaS_2$ (S2e), $GeS_2|SnS_2$ (S2f), $SnSe_2|ZrTe_2$ (S2g), $HfS_2|ZrS_2$ (S2h), and $MoS_2|SnS_2$ (S2i).

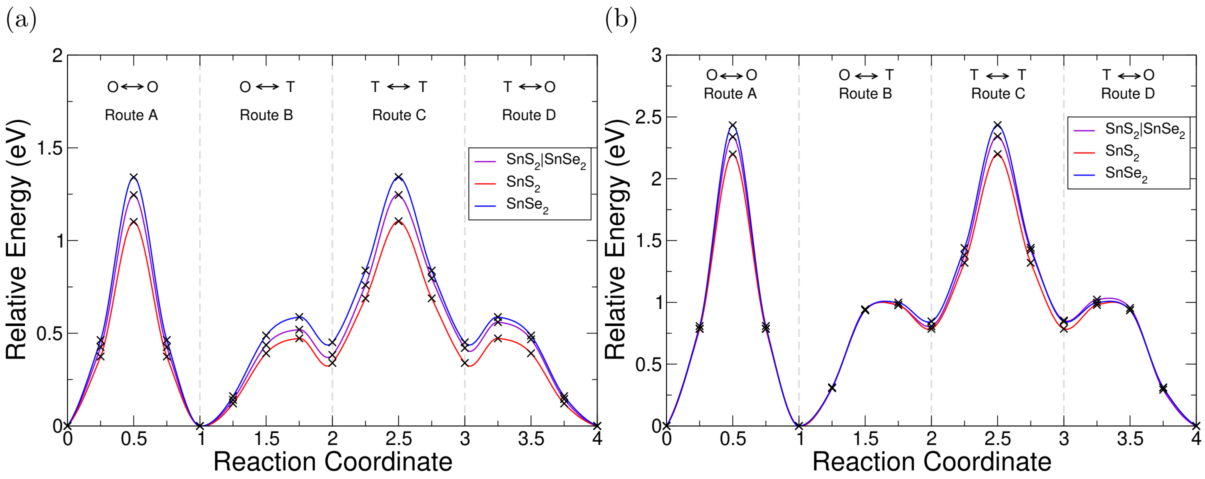

Figure S3: Nudged elastic band diffusion barriers for lithium (S3a) and magnesium (S3b) along unique routes in $SnS_2|SnSe_2$ (purple), SnS_2 (red), and $SnSe_2$ (blue). In the individual TMDCs, Route B and Route D are equivalent.

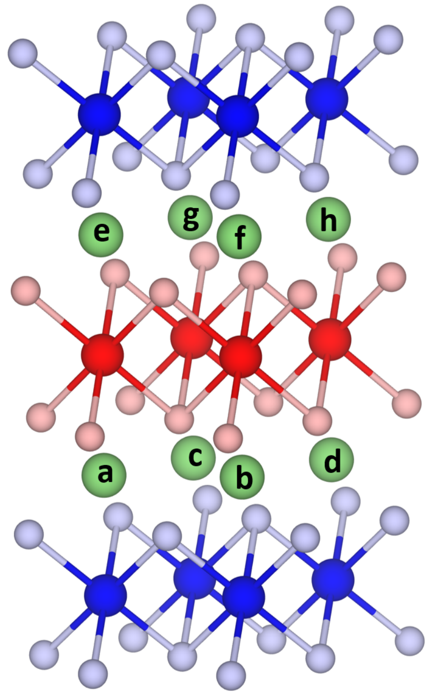

Figure S4: The different intercalation sites available in the TMDC superlattices considered in this work, indexed a-h.

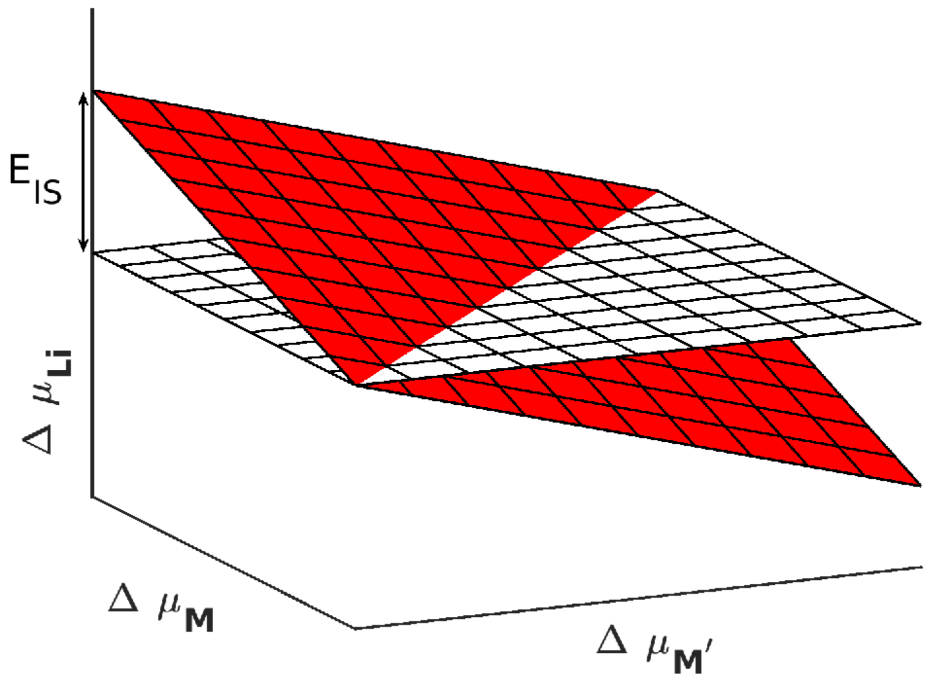

Figure S5: Schematic of a phase diagram for a superlattice structure. The horizontal white plane is described by equation (S11), and the diagonal red plane is described by equation (S13). E_{IS} is indicated.

Phase Diagram Derivation

Here, we have generalised the approach of assessing intercalation stability of TMDCs[12] to superlattice structures which consist of alternating layers of lattice-matched TMDCs, to determine an equivalent expression of E_{IS} for superlattice systems. For an arbitrary (lattice-matched) superlattice of two TMDC materials (SL = $MX_2M'X'_2$) when intercalated with lithium ($Li_bSL = Li_bMX_2M'X'_2$) we define the enthalpy of formation of relevant products:

$$\Delta H(\mathrm{Li_bSL}) = E(\mathrm{Li_bSL}) - [b\mu^0_{Li} + \mu^0_M + \mu^0_{M'} + 2\mu^0_X + 2\mu^0_{X'}] \tag{S1}$$

$$\Delta H(\mathrm{SL}) = E(\mathrm{SL}) - [\mu^0_M + \mu^0_{M'} + 2\mu^0_X + 2\mu^0_{X'}], \tag{S2}$$

$$\Delta H(\mathrm{Li_2X}) = E(\mathrm{Li_2X}) - [2\mu^0_{Li} + \mu^0_X], \tag{S3}$$

$$\Delta H(\mathrm{Li_2X'}) = E(\mathrm{Li_2X'}) - [2\mu^0_{Li} + \mu^0_{X'}] \tag{S4}$$

where $\Delta H(A)$ gives the enthalpy of formation of the compound A, $E(A)$ gives the energy of the compound A, and $\mu^0_B = E(B)$ gives the chemical potential of elemental species B when it is in its elemental bulk structure.

The thermodynamic equilibrium condition requires,

$$\Delta H(\mathrm{Li_bSL}) = b\Delta\mu_{Li} + \Delta\mu_M + \Delta\mu_{M'} + 2\Delta\mu_X + 2\Delta\mu_{X'}, \tag{S5}$$

where we have used the notation $\Delta\mu_B = \mu_B - \mu^0_B$, with μ_B being the chemical potential of elemental species B in $\mathrm{Li_bSL}$. This simply states that the energy of the superlattice structure is the sum of the chemical potentials of the constituent atoms. Rearranging the thermodynamic equilibrium condition gives,

$$\Delta\mu_X + \Delta\mu_{X'} = \frac{1}{2}\big\{\Delta H(\mathrm{Li_bSL}) - [b\Delta\mu_{Li} + \Delta\mu_M + \Delta\mu_{M'}]\big\}. \tag{S6}$$

We require that $\mathrm{Li_2X}$, $\mathrm{Li_2X'}$, $\mathrm{MX_2M'X'_2}$ and the bulk forms of the component elements do not form. For superlattices in particular, it is unreasonable to assume that the layered structure would be obtained upon reversal of the conversion reaction. Thermodynamic phase diagrams can therefore be constructed by imposing limits on the chemical potentials of the component elements,

$$\Delta\mu_M + \Delta\mu_{M'} + 2\Delta\mu_X + 2\Delta\mu_{X'} \leq \Delta H(\mathrm{SL}), \tag{S7}$$

$$2\Delta\mu_{Li} + \Delta\mu_{X} \leq \Delta H(\mathrm{Li_2X}), \tag{S8}$$

$$2\Delta\mu_{Li} + \Delta\mu_{X'} \leq \Delta H(\mathrm{Li_2X'}), \tag{S9}$$

and,

$$\Delta\mu_{Li,M,M',X,X'} \leq 0. \tag{S10}$$

Substituting (S6) into (S7) and rearranging results in,

$$\frac{1}{b}\big\{\Delta H(\mathrm{Li_bSL}) - \Delta H(\mathrm{SL})\big\} \leq \Delta\mu^{(2)}_{Li}. \tag{S11}$$

This then gives the thermodynamic limit on the lithium chemical potential such that the intercalation of the superlattice is preferred to the pristine superlattice and bulk lithium. We now add equations (S8) and (S9) to get,

$$4\Delta\mu_{Li} \leq \Delta H(\mathrm{Li_2X}) + \Delta H(\mathrm{Li_2X'}) - \Delta\mu_{X} - \Delta\mu_{X'}, \tag{S12}$$

and make use of equation (S6) to get,

$$\Delta\mu^{(1)}_{Li} \leq \frac{1}{8-b}\big[2\Delta H(\mathrm{Li_2X}) + 2\Delta H(\mathrm{Li_2X'}) - \Delta H(\mathrm{Li_bSL}) + \Delta\mu_{M} + \Delta\mu_{M'}\big]. \tag{S13}$$

This is then the thermodynamic limit on the chemical potential of the lithium so that the conversion-reaction products $\mathrm{Li_2X}$ and $\mathrm{Li_2X'}$ do not form.

Each of $\Delta\mu^{(1)}_{Li}$ and $\Delta\mu^{(2)}_{Li}$ define a plane, analogous to the one-dimensional boundaries for individual TMDCs. The first of these boundaries is a diagonal plane, as indicated by the red and black plane in Figure S5, and the second defines flat/constant plane, as indicated by the white and black plane in Figure S5. These limits on the appropriate chemical potentials

ensure stability of the intercalated superlattice structure against decomposition into the experimentally observed Li_2X crystals, the elemental bulk structures, and pristine superlattice SL, respectively. Equivalent expressions can be obtained for magnesium-intercalation, for compounds Mg_bSL, MgX, and MgX′.

We can then evaluate the difference between the intercepts of planes 1 and 2 with the $\Delta\mu_{Li}$-axis, defining a quantity E_{IS} that can be used to compare different phase diagrams,

$$E_{IS} = \Delta\mu_{Li}^{(1)}(\Delta\mu_{M,M'} = 0) - \Delta\mu_{Li}^{(2)}(\Delta\mu_{M,M'} = 0). \tag{S14}$$

Here, $\Delta\mu_{Li}^{(1/2)}(\Delta\mu_{M,M'} = 0)$ is the value of the boundary plane 1/2 at the point where $\Delta\mu_{M,M'} = 0$. This gives,

$$E_{IS}^{\mathrm{Li}} = \frac{2}{8-b}\left[\Delta H(\mathrm{Li_2X}) + \Delta H(\mathrm{Li_2X'})\right] + \frac{1}{b}\Delta H(\mathrm{SL}) - \frac{8}{8b-b^2}\Delta H(\mathrm{Li_bSL}). \tag{S15}$$

Each of the enthalpy of formation values should be negative for them to be thermodynamically stable with respect to their atomic constituents. When the value of E_{IS} is negative, the first two terms dominate, and plane 1 intercepts below plane 2 so no stability region exists. When the value of E_{IS} is positive, however, $\Delta H(\mathrm{Li}_b\mathrm{SL})$ dominates and the intercalated SL material is stable. For magnesium intercalation, we have an equivalent expression,

$$E_{IS}^{\mathrm{Mg}} = \frac{2}{4-b}\left[\Delta H(\mathrm{MgX}) + \Delta H(\mathrm{MgX'})\right] + \frac{1}{b}\Delta H(\mathrm{SL}) - \frac{4}{4b-b^2}\Delta H(\mathrm{Mg}_b\mathrm{SL}). \tag{S16}$$

In the case where the two component TMDCs are the same (i.e. M = M′, X = X′, $b = 2a$, $\mathrm{Li_bMX_2M'X'_2} = \mathrm{Li_aMX_2}$), all of the expressions presented above simplify to those for the individual TMDCs.[12] A schematic phase diagram for a single TMDC and a superlattice TMDC using these limits is then presented in Figure S6 and Figure S7 .

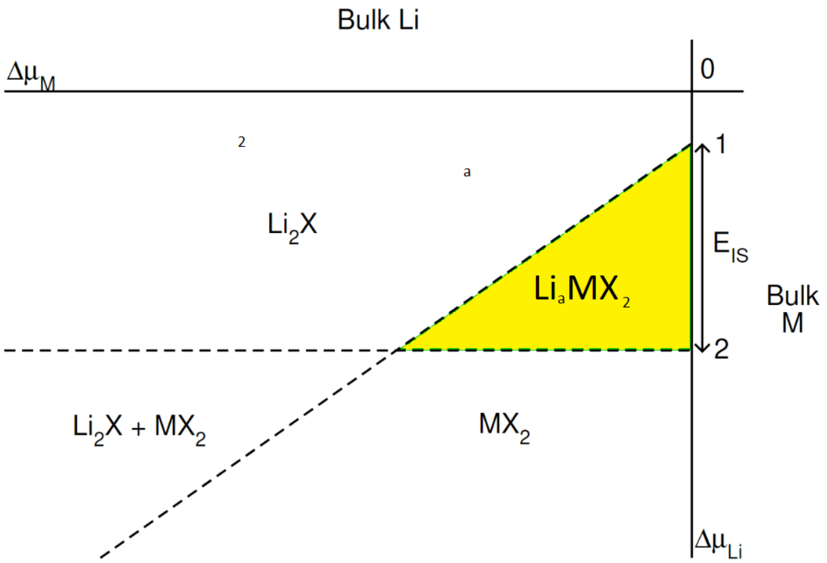

Figure S6: Schematic of a phase diagram for an individual TMDC. The horizontal line describes the boundary between the pristine and intercalated structures (analogous to equation (S11)), and the diagonal describes the boundary between the intercalated TMDC and the Li_2X conversion product (analogous to equation (S13)). The yellow-shaded region indicates the window of stability, and the corresponding E_{IS} is indicated.

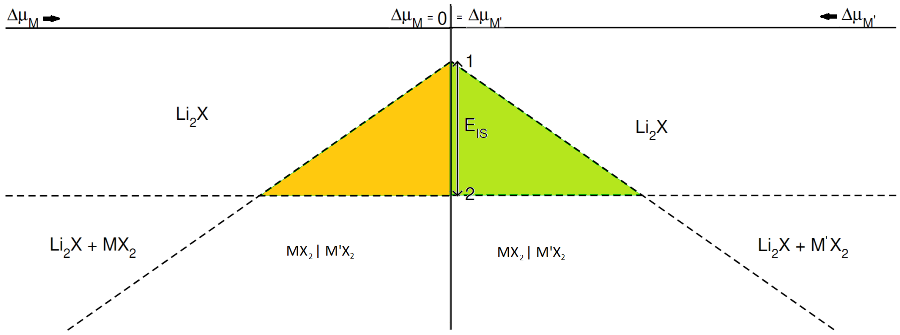

Figure S7: Schematic of a phase diagram for a TMDC superlattice presenting the 3D plot of S5 in 2D. The yellow/green-shaded regions indicates the window of stability, and the corresponding E_{IS} is indicated. For the positive x-axis, the chemical depends on the M' metal and for the negative x-axis the chemical potential depends on the M metal.

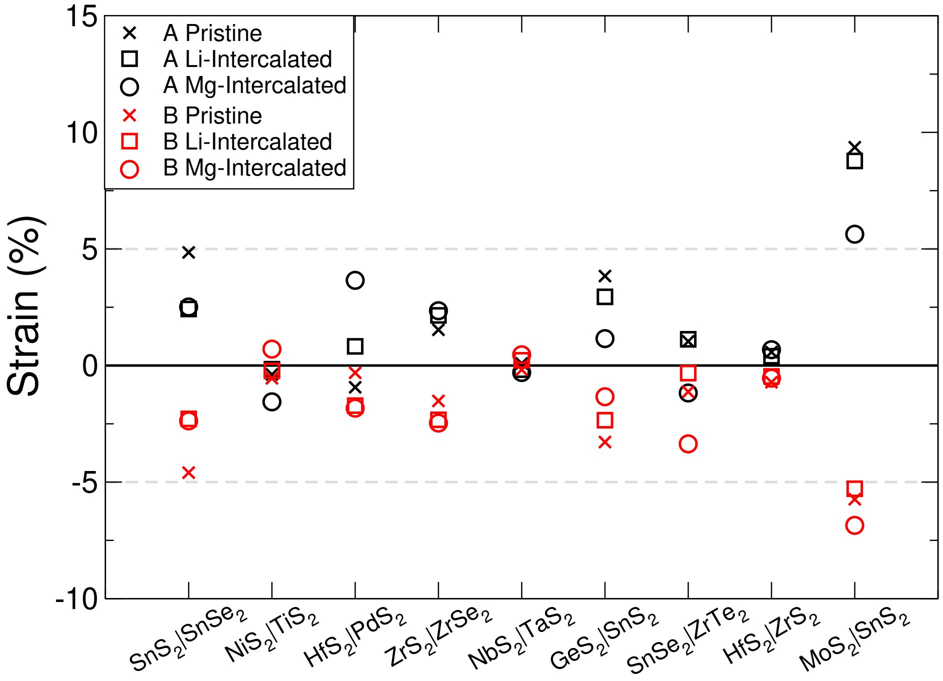

Figure S8: Resultant strains on the component TMDCs when combined in the superlattice structure. For a superlattice of the form $MX_2|M'X'_2 = A|B$, the strain on A is given by $\frac{a_{SL}^{P/I} - a_{A}^{P/I}}{a_{A}^{P/I}} \times 100$.

Superlattice Construction

In this section, we investigate the construction of a superlattice without any intercalants.

Lattice Matching

To minimise the strain on each of the component materials comprising a superlattice, a close matching of each of the respective in-plane lattice constants is required. Here, we evaluate how well-matched different pairings are when combined in a superlattice. We focus on selected systems $SnS_2|SnSe_2$, $NiS_2|TiS_2$, $HfS_2|PdS_2$, $ZrS_2|ZrSe_2$, $NbS_2|TaS_2$, $GeS_2|SnS_2$, $SnSe_2|ZrTe_2$, $HfS_2|ZrS_2$, and $MoS_2|SnS_2$. In Figure S8 we see the percentage strain on the pristine TMDCs when at the lattice constant of the formed superlattice without intercalants. For component A (with lattice constant a_A^P) strained to the lattice constant of the superlattice (a_{SL}^P), the strain is calculated using $\frac{a_{SL}^P - a_A^P}{a_A^P} \times 100$. Similarly, we also present the percentage

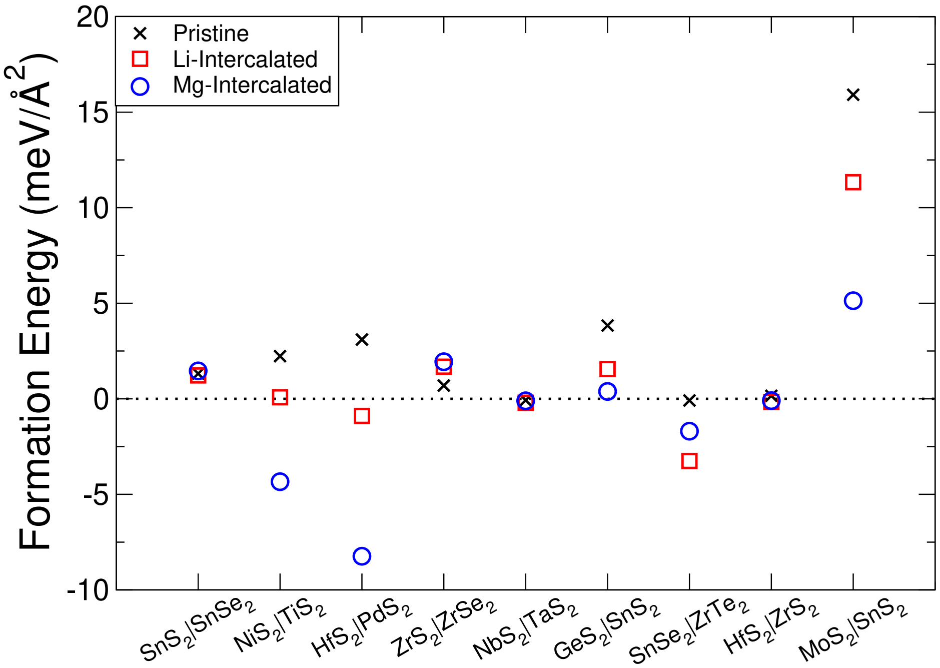

Figure S9: Formation energy of pristine and intercalated superlattice from the pristine and intercalated components, respectively. Calculated using $E_{form} = [E_{SL}^{P/I} - (E_A^{P/I} + E_B^{P/I})]/S$.

strain on the intercalated TMDCs when at the lattice constant of the intercalated superlattice. For component A of lattice constant a_A^I strained to the lattice constant a_{SL}^I, the strain is calculated using $\frac{a_{SL}^I - a_A^I}{a_A^I} \times 100$. With the exception of $MoS_2|SnS_2$, all TMDCs show strains of less than 5%, indicating that the paired systems are well lattice-matched. The $MoS_2|SnS_2$ pairing has been included to see if larger values of strain have significant effects on the determined properties of superlattices. As we find the trends of the other TMDCs highlighted in the main article to be present in the $MoS_2|SnS_2$ pairing, we conclude that moderate strains (and hence superlattices with larger unit cell mismatches) do not significantly affect the results.

Formation Energy

We evaluate the energy (per formula unit) required to form the superlattice, in both the pristine and intercalated systems. This has been calculated using $E_{form} = [E_{SL}^{P/I} - (E_A^{P/I} + E_B^{P/I})]/S$, with interface surface area S, energies of the superlattice, $E_{SL}^{P/I}$, and component

Table S3: In-plane lattice constants of TMDCs and formed superlattices, as well as the resultant strains on the component TMDCs when combined in the superlattice structure. The lattice constants are for the 2×2 supercells used. Data presented is for the pristine form of the superlattice and TMDC. For a superlattice of the form $MX_2|M'X'_2$ = A|B, the strain on A is given by $\frac{a^P_{SL} - a^P_A}{a^P_A} \times 100$.

Superlattice		Component A		Component B	
A|B	a (Å)	a (Å)	Strain (%)	a (Å)	Strain (%)
CuS_2|OsS_2	6.90	6.96	-0.86	6.96	-0.83
CuS_2|RhS_2	6.96	6.96	0.00	6.97	-0.05
GeS_2|SnS_2	7.12	6.86	3.84	7.36	-3.28
GeS_2|$SnSe_2$	7.30	6.86	6.38	7.72	-5.48
GeS_2|$TiSe_2$	6.94	6.86	1.14	7.05	-1.59
$GeSe_2$|HfS_2	7.23	7.24	-0.16	7.23	-0.02
$GeSe_2$|$NiSe_2$	7.31	7.24	0.96	7.17	1.84
$GeSe_2$|$SnSe_2$	7.50	7.24	3.57	7.72	-2.90
$GeSe_2$|$TaTe_2$	7.31	7.24	1.00	7.30	0.07
$GeSe_2$|$TiSe_2$	7.16	7.24	-1.12	7.05	1.53
$GeSe_2$|$TiTe_2$	7.37	7.24	1.81	7.49	-1.65
HfS_2|PdS_2	7.16	7.23	-0.93	7.18	-0.31
HfS_2|PtS_2	7.18	7.23	-0.68	7.20	-0.32
HfS_2|SnS_2	7.29	7.23	0.86	7.36	-1.01
HfS_2|$SnSe_2$	7.45	7.23	3.09	7.72	-3.49
HfS_2|ZrS_2	7.27	7.23	0.58	7.32	-0.72
$HfTe_2$|$PbSe_2$	7.91	7.81	1.32	8.51	-7.00
$HfTe_2$|$SnSe_2$	7.77	7.81	-0.59	7.72	0.59
MoS_2|SnS_2	6.94	6.35	9.36	7.36	-5.74
MoS_2|VS_2	6.34	6.35	-0.10	6.33	0.19
MoS_2|WS_2	6.36	6.35	0.22	6.38	-0.32
MoS_2|WSe_2	6.46	6.35	1.73	6.54	-1.20
$MoTe_2$|OsS_2	7.05	6.94	1.44	6.96	1.21
NbS_2|TaS_2	6.68	6.68	0.09	6.70	-0.18

Table S4: In-plane lattice constants of TMDCs and formed superlattices, as well as the resultant strains on the component TMDCs when combined in the superlattice structure. The lattice constants are for the 2 × 2 supercells used. Data presented is for the pristine form of the superlattice and TMDC. For a superlattice of the form $MX_2|M'X'_2$ = A|B, the strain on A is given by $\frac{a^P_{SL} - a^P_A}{a^P_A} \times 100$.

Superlattice		Component A		Component B	
A|B	a (Å)	a (Å)	Strain (%)	a (Å)	Strain (%)
$NiS_2\|SnS_2$	7.05	6.77	4.05	7.36	-4.27
$NiS_2\|TiS_2$	6.75	6.77	-0.38	6.79	-0.55
$NiTe_2\|PbSe_2$	7.95	7.74	2.69	8.51	-6.60
$NiTe_2\|SnSe_2$	7.84	7.74	1.28	7.72	1.55
$OsS_2\|RhS_2$	6.93	6.96	-0.42	6.97	-0.50
$OsTe_2\|SnSe_2$	7.87	7.76	1.48	7.72	2.01
$PbS_2\|PbSe_2$	8.27	8.04	2.90	8.51	-2.78
$PbS_2\|YS_2$	7.94	8.04	-1.22	8.14	-2.38
$PbSe_2\|SnTe_2$	8.69	8.51	2.09	8.74	-0.60
$PbSe_2\|ZrTe_2$	7.96	8.51	-6.49	7.89	0.88
$PdS_2\|PtS_2$	7.20	7.18	0.23	7.20	-0.03
$PtS_2\|ZrS_2$	7.22	7.20	0.26	7.32	-1.38
$ScS_2\|SnS_2$	7.37	7.42	-0.76	7.36	0.05
$ScS_2\|TiTe_2$	7.36	7.42	-0.81	7.49	-1.71
$ScTe_2\|SnSe_2$	7.83	7.90	-0.94	7.72	1.44
$SnS_2\|SnSe_2$	7.54	7.36	2.43	7.72	-2.30
$SnS_2\|TiSe_2$	7.23	7.36	-1.78	7.05	2.60
$SnS_2\|TiTe_2$	7.41	7.36	0.67	7.49	-1.06
$SnS_2\|ZrS_2$	7.34	7.36	-0.32	7.32	0.25
$SnS_2\|ZrSe_2$	7.46	7.36	1.34	7.55	-1.15
$SnSe_2\|TiTe_2$	7.62	7.72	-1.25	7.49	1.75
$SnSe_2\|ZrSe_2$	7.63	7.72	-1.19	7.55	1.05
$SnSe_2\|ZrTe_2$	7.80	7.72	1.04	7.89	-1.13
$VS_2\|WS_2$	6.35	6.33	0.40	6.38	-0.42
$ZrS_2\|ZrSe_2$	7.43	7.32	1.53	7.55	-1.52

Table S5: In-plane lattice constants of TMDCs and formed superlattices, as well as the resultant strains on the component TMDCs when combined in the superlattice structure. The lattice constants are for the 2 × 2 supercells used. Data presented is for the lithium-intercalated form of the superlattice and TMDC. For a superlattice of the form $MX_2|M'X'_2 = A|B$, the strain on A is given by $\frac{a^{Li}_{SL} - a^{Li}_{A}}{a^{Li}_{A}} \times 100$.

Superlattice		Component A		Component B	
A|B	a (Å)	a (Å)	Strain (%)	a (Å)	Strain (%)
$CuS_2\|OsS_2$	7.09	6.98	1.63	7.21	-1.72
$CuS_2\|RhS_2$	7.05	6.98	1.09	7.15	-1.39
$GeS_2\|SnS_2$	7.31	7.10	2.95	6.80	7.55
$GeS_2\|SnSe_2$	7.52	7.10	5.81	7.85	-4.25
$GeS_2\|TiSe_2$	7.16	7.10	0.73	7.18	-0.30
$GeSe_2\|HfS_2$	7.27	7.50	-3.05	7.02	3.57
$GeSe_2\|NiSe_2$	7.38	7.50	-1.55	7.26	1.72
$GeSe_2\|SnSe_2$	7.69	7.50	2.53	7.85	-2.05
$GeSe_2\|TaTe_2$	7.56	7.50	0.75	7.47	1.12
$GeSe_2\|TiSe_2$	7.35	7.50	-2.06	7.18	2.34
$GeSe_2\|TiTe_2$	7.65	7.50	2.06	7.84	-2.34
$HfS_2\|PdS_2$	7.08	7.02	0.82	7.20	-1.72
$HfS_2\|PtS_2$	7.12	7.02	1.36	7.29	-2.42
$HfS_2\|SnS_2$	7.27	7.02	3.55	6.80	6.90
$HfS_2\|SnSe_2$	7.43	7.02	5.84	7.85	-5.35
$HfS_2\|ZrS_2$	7.05	7.02	0.40	7.08	-0.47
$HfTe_2\|PbSe_2$	8.01	7.91	1.19	8.03	-0.30
$HfTe_2\|SnSe_2$	7.92	7.91	0.05	7.85	0.84
$MoS_2\|SnS_2$	7.09	6.52	8.77	6.80	4.31
$MoS_2\|VS_2$	6.55	6.52	0.48	6.58	-0.37
$MoS_2\|WS_2$	6.50	6.52	-0.25	6.48	0.33
$MoS_2\|WSe_2$	6.60	6.52	1.24	6.76	-2.39
$MoTe_2\|OsS_2$	7.22	7.09	1.79	7.21	0.05
$NbS_2\|TaS_2$	6.69	6.70	-0.18	6.67	0.23

Table S6: In-plane lattice constants of TMDCs and formed superlattices, as well as the resultant strains on the component TMDCs when combined in the superlattice structure. The lattice constants are for the 2 × 2 supercells used. Data presented is for the lithium-intercalated form of the superlattice and TMDC. For a superlattice of the form $MX_2|M'X'_2$ = A|B, the strain on A is given by $\frac{a_{SL}^{Li} - a_A^{Li}}{a_A^{Li}} \times 100$.

Superlattice		Component A		Component B	
A|B	a (Å)	a (Å)	Strain (%)	a (Å)	Strain (%)
$NiS_2|SnS_2$	7.15	6.80	5.09	7.49	-4.58
$NiS_2|TiS_2$	6.79	6.80	-0.16	6.80	-0.23
$NiTe_2|PbSe_2$	8.05	7.96	1.05	8.03	0.21
$NiTe_2|SnSe_2$	7.91	7.96	-0.62	7.85	0.80
$OsS_2|RhS_2$	7.19	7.21	-0.35	7.15	0.52
$OsTe_2|SnSe_2$	8.15	8.24	-1.02	7.85	3.85
$PbS_2|PbSe_2$	7.85	7.68	2.23	8.03	-2.21
$PbS_2|YS_2$	7.64	7.68	-0.53	7.62	0.32
$PbSe_2|SnTe_2$	8.24	8.03	2.57	8.44	-2.37
$PbSe_2|ZrTe_2$	8.03	8.03	0.06	7.96	0.88
$PdS_2|PtS_2$	7.25	7.20	0.62	7.29	-0.63
$PtS_2|ZrS_2$	7.15	7.29	-2.00	7.08	0.92
$ScS_2|SnS_2$	7.33	7.22	1.54	7.49	-2.09
$ScS_2|TiTe_2$	7.41	7.22	2.59	7.84	-5.48
$ScTe_2|SnSe_2$	7.99	8.19	-2.48	7.85	1.78
$SnS_2|SnSe_2$	7.67	7.49	2.43	7.85	-2.29
$SnS_2|TiSe_2$	7.34	7.49	-2.05	7.18	2.20
$SnS_2|TiTe_2$	7.61	7.49	1.63	7.84	-2.90
$SnS_2|ZrS_2$	7.29	7.49	-2.64	7.08	2.97
$SnS_2|ZrSe_2$	7.48	7.49	-0.18	7.41	0.94
$SnSe_2|TiTe_2$	7.83	7.85	-0.30	7.84	-0.14
$SnSe_2|ZrSe_2$	7.64	7.85	-2.68	7.41	3.15
$SnSe_2|ZrTe_2$	7.94	7.85	1.12	7.96	-0.32
$VS_2|WS_2$	6.54	6.58	-0.55	6.48	0.89
$ZrS_2|ZrSe_2$	7.23	7.08	2.15	7.41	-2.33

Table S7: In-plane lattice constants of TMDCs and formed superlattices, as well as the resultant strains on the component TMDCs when combined in the superlattice structure. The lattice constants are for the 2×2 supercells used. Data presented is for the magnesium-intercalated form of the superlattice and TMDC. For a superlattice of the form $MX_2|M'X'_2 = A|B$, the strain on A is given by $\frac{a_{SL}^{Mg} - a_{A}^{Mg}}{a_{A}^{Mg}} \times 100$.

Superlattice		Component A		Component B	
A|B	a (Å)	a (Å)	Strain (%)	a (Å)	Strain (%)
CuS_2|OsS_2	7.35	7.16	2.73	7.49	-1.77
CuS_2|RhS_2	7.30	7.16	1.99	7.32	-0.25
GeS_2|SnS_2	7.55	7.46	1.15	7.12	5.94
GeS_2|$SnSe_2$	7.73	7.46	3.66	8.03	-3.71
GeS_2|$TiSe_2$	7.45	7.46	-0.18	7.40	0.61
$GeSe_2$|HfS_2	7.44	7.83	-5.03	6.93	7.33
$GeSe_2$|$NiSe_2$	7.64	7.83	-2.45	7.48	2.18
$GeSe_2$|$SnSe_2$	7.93	7.83	1.21	8.03	-1.28
$GeSe_2$|$TaTe_2$	7.88	7.83	0.64	8.39	-5.99
$GeSe_2$|$TiSe_2$	7.65	7.83	-2.34	7.40	3.36
$GeSe_2$|$TiTe_2$	8.01	7.83	2.26	8.20	-2.27
HfS_2|PdS_2	7.19	6.93	3.66	7.32	-1.83
HfS_2|PtS_2	7.20	6.93	3.87	7.42	-3.00
HfS_2|SnS_2	7.23	6.93	4.31	7.12	1.50
HfS_2|$SnSe_2$	7.41	6.93	6.91	8.03	-7.73
HfS_2|ZrS_2	6.98	6.93	0.68	7.02	-0.54
$HfTe_2$|$PbSe_2$	7.88	8.35	-5.65	8.11	-2.83
$HfTe_2$|$SnSe_2$	7.87	8.35	-5.74	8.03	-2.03
MoS_2|SnS_2	7.13	6.75	5.63	7.12	0.02
MoS_2|VS_2	7.03	6.75	4.26	6.88	2.19
MoS_2|WS_2	6.65	6.75	-1.45	6.54	1.65
MoS_2|WSe_2	6.71	6.75	-0.53	6.72	-0.13
$MoTe_2$|OsS_2	7.80	7.49	4.14	7.49	4.14
NbS_2|TaS_2	6.75	6.78	-0.30	6.72	0.46

Table S8: In-plane lattice constants of TMDCs and formed superlattices, as well as the resultant strains on the component TMDCs when combined in the superlattice structure. The lattice constants are for the 2×2 supercells used. Data presented is for the magnesium-intercalated form of the superlattice and TMDC. For a superlattice of the form $MX_2|M'X'_2 =$ A|B, the strain on A is given by $\frac{a_{SL}^{Mg} - a_A^{Mg}}{a_A^{Mg}} \times 100$.

Superlattice		Component A		Component B	
A|B	a (Å)	a (Å)	Strain (%)	a (Å)	Strain (%)
NiS_2|SnS_2	7.32	7.12	2.68	7.65	-4.37
NiS_2|TiS_2	7.01	7.12	-1.56	6.96	0.70
$NiTe_2$|$PbSe_2$	7.96	8.16	-2.43	8.11	-1.75
$NiTe_2$|$SnSe_2$	7.94	8.16	-2.68	8.03	-1.10
OsS_2|RhS_2	7.43	7.49	-0.80	7.32	1.47
$OsTe_2$|$SnSe_2$	8.39	8.57	-2.10	8.03	4.50
PbS_2|$PbSe_2$	7.92	7.73	2.42	8.11	-2.28
PbS_2|YS_2	7.51	7.73	-2.88	7.40	1.51
$PbSe_2$|$SnTe_2$	8.39	8.11	3.49	8.67	-3.24
$PbSe_2$|$ZrTe_2$	7.94	8.11	-2.10	8.21	-3.37
PdS_2|PtS_2	7.39	7.32	0.96	7.42	-0.44
PtS_2|ZrS_2	7.22	7.42	-2.77	7.02	2.85
ScS_2|SnS_2	7.32	7.20	1.70	7.65	-4.32
ScS_2|$TiTe_2$	7.50	7.20	4.24	8.20	-8.49
$ScTe_2$|$SnSe_2$	8.10	8.29	-2.29	8.03	0.79
SnS_2|$SnSe_2$	7.84	7.65	2.51	8.03	-2.37
SnS_2|$TiSe_2$	7.37	7.65	-3.62	7.40	-0.40
SnS_2|$TiTe_2$	7.58	7.65	-0.97	8.20	-7.59
SnS_2|ZrS_2	7.27	7.65	-5.01	7.02	3.55
SnS_2|$ZrSe_2$	7.46	7.65	-2.52	7.36	1.26
$SnSe_2$|$TiTe_2$	7.94	8.03	-1.10	8.20	-3.10
$SnSe_2$|$ZrSe_2$	7.66	8.03	-4.69	7.36	3.96
$SnSe_2$|$ZrTe_2$	7.94	8.03	-1.18	8.21	-3.36
VS_2|WS_2	6.78	6.88	-1.55	6.54	3.61
ZrS_2|$ZrSe_2$	7.18	7.02	2.35	7.36	-2.47

TMDC materials, $E_A^{P/I}$ and $E_B^{P/I}$. Positive values of E_{form} indicate superlattices where it costs energy to combine the components, whereas negative values indicate superlattices in which it is energetically preferred for the components to be combined rather than exist as their respective bulk materials. These results are presented in Figure S9. Formation energies for most of the systems are small (remaining below 5 meV/Å^2), with several structures demonstrating negative formation energy. We generally see that systems with lower strains result in more favourable formation energies, resulting in the most strained system (MoS_2|SnS_2) demonstrating the highest formation energy. Interestingly, the formation energy is reduced with the introduction of an intercalant, as highlighted with the NiS_2|TiS_2 system which has a formation energy of 2.23 meV/Å^2, which is reduced to 0.08 meV/Å^2 with lithium intercalation, and to -4.34 meV/Å^2 with magnesium intercalation. Thus, the introduction of an intercalant can stabilise the superlattice, and lead to superlattice construction being an energetically downhill process.

Intercalation of Superlattices

Here, we assess the effect that superlattice construction has on the key electrode properties. First, we present the volumetric expansion arising from intercalation. Then we consider the intercalation voltage and the thermodynamic stability with E_{IS}. We then present the electronic structure of selected systems and show how they evolve with intercalation, and present the elastic properties.

Volumetric Expansion

As we discussed in the main article, the volumetric expansion that arises from intercalation is an important factor that must be considered for any potential electrode material. In Table S9 and Table S10 we show the volumetric expansion for the investigated superlattices as they are intercalated with lithium from $MX_2M'X_2$ to $Li_2MX_2M'X_2$. Similarly, Table S11 and Table S12 we show the volumetric expansion arising from magnesium intercalation. These have each been calculated using $\frac{V-V_0}{V_0} \times 100$ for initial volume V_0 and final volume V. We have also included the corresponding expansions for the component TMDCs[12] for easy comparison. From this, we conclude that the average volumetric expansion of the component TMDCs provides a good estimate for the volumetric expansion that arises in the formed superlattice.

Table S9: Superlattice and component volumetric expansion for the considered superlattices intercalated with lithium.

Superlattice A|B	Superlattice Expansion (%)	Component A Expansion (%)	Component B Expansion (%)
CuS_2|OsS_2	24.87	29.39	15.81
CuS_2|RhS_2	28.18	29.39	23.70
GeS_2|SnS_2	11.15	12.15	10.17
GeS_2|$SnSe_2$	12.52	12.15	10.02
GeS_2|$TiSe_2$	11.86	12.15	9.74
$GeSe_2$|HfS_2	7.13	12.41	0.32
$GeSe_2$|$NiSe_2$	22.48	12.41	26.67
$GeSe_2$|$SnSe_2$	11.98	12.41	10.02
$GeSe_2$|$TaTe_2$	16.26	12.41	13.66
$GeSe_2$|$TiSe_2$	11.08	12.41	9.74
$GeSe_2$|$TiTe_2$	15.66	12.41	14.80
HfS_2|PdS_2	7.80	0.32	16.54
HfS_2|PtS_2	7.69	0.32	23.58
HfS_2|SnS_2	6.76	0.32	10.17
HfS_2|$SnSe_2$	6.26	0.32	10.02
HfS_2|ZrS_2	0.27	0.32	0.31
$HfTe_2$|$PbSe_2$	13.38	7.38	17.93
$HfTe_2$|$SnSe_2$	10.72	7.38	10.02
MoS_2|SnS_2	11.02	15.06	10.17
MoS_2|VS_2	12.04	15.06	12.26
MoS_2|WS_2	10.86	15.06	12.87
MoS_2|WSe_2	11.82	15.06	14.35
$MoTe_2$|OsS_2	19.36	14.22	15.81
NbS_2|TaS_2	7.86	10.93	9.04

Table S10: Superlattice and component volumetric expansion for the considered superlattices intercalated with lithium.

Superlattice A|B	Superlattice Expansion (%)	Component A Expansion (%)	Component B Expansion (%)
NiS_2|SnS_2	11.55	26.67	10.17
NiS_2|TiS_2	14.79	26.67	6.61
$NiTe_2$|$PbSe_2$	20.13	21.35	17.93
$NiTe_2$|$SnSe_2$	20.44	21.35	10.02
OsS_2|RhS_2	21.27	15.81	23.70
$OsTe_2$|$SnSe_2$	18.12	14.08	10.02
PbS_2|$PbSe_2$	18.83	19.77	17.93
PbS_2|YS_2	13.89	19.77	5.21
$PbSe_2$|$SnTe_2$	17.77	17.93	17.30
$PbSe_2$|$ZrTe_2$	13.38	17.93	7.96
PtS_2|ZrS_2	8.07	23.58	0.31
ScS_2|SnS_2	10.76	8.42	10.17
ScS_2|$TiTe_2$	15.71	8.42	14.80
$ScTe_2$|$SnSe_2$	13.97	13.16	10.02
SnS_2|$SnSe_2$	10.92	10.17	10.02
SnS_2|$TiSe_2$	9.58	10.17	9.74
SnS_2|$TiTe_2$	12.44	10.17	14.80
SnS_2|ZrS_2	6.02	10.17	0.31
SnS_2|$ZrSe_2$	8.36	10.17	2.17
$SnSe_2$|$TiTe_2$	11.68	10.02	14.80
$SnSe_2$|$ZrTe_2$	10.92	10.02	7.96
VS_2|WS_2	11.00	12.26	12.87
ZrS_2|$ZrSe_2$	1.18	0.31	2.175

Table S11: Superlattice and component volumetric expansion for the considered superlattices intercalated with magnesium.

Superlattice A|B	Superlattice Expansion (%)	Component A Expansion (%)	Component B Expansion (%)
CuS_2|OsS_2	35.07	42.66	24.38
CuS_2|RhS_2	39.16	42.66	32.07
GeS_2|SnS_2	23.07	26.56	26.56
GeS_2|$SnSe_2$	23.20	26.56	24.40
GeS_2|$TiSe_2$	21.17	26.56	15.65
$GeSe_2$|HfS_2	13.11	24.40	2.01
$GeSe_2$|$NiSe_2$	33.91	24.40	35.42
$GeSe_2$|$SnSe_2$	22.80	24.40	24.40
$GeSe_2$|$TaTe_2$	24.39	24.40	22.08
$GeSe_2$|$TiSe_2$	19.81	24.40	15.65
$GeSe_2$|$TiTe_2$	24.03	24.40	19.84
HfS_2|PdS_2	14.18	2.01	34.62
HfS_2|PtS_2	12.86	2.01	30.95
HfS_2|SnS_2	15.27	2.01	26.56
HfS_2|$SnSe_2$	12.45	2.01	24.40
HfS_2|ZrS_2	1.92	2.01	2.04
$HfTe_2$|$PbSe_2$	20.03	12.98	26.27
$HfTe_2$|$SnSe_2$	21.81	12.98	24.40
MoS_2|SnS_2	20.36	19.95	26.56
MoS_2|VS_2	25.49	19.95	21.50
MoS_2|WS_2	17.81	19.95	15.97
MoS_2|WSe_2	18.56	19.95	18.57
$MoTe_2$|OsS_2	28.52	16.80	24.38
NbS_2|TaS_2	12.87	13.93	11.36

Table S12: Superlattice and component volumetric expansion for the considered superlattices intercalated with magnesium.

Superlattice A|B	Superlattice Expansion (%)	Component A Expansion (%)	Component B Expansion (%)
NiS_2|SnS_2	21.64	42.10	26.56
NiS_2|TiS_2	23.32	42.10	12.21
$NiTe_2$|$PbSe_2$	30.34	32.62	26.27
$NiTe_2$|$SnSe_2$	30.44	32.62	24.40
OsS_2|RhS_2	29.32	24.38	32.07
$OsTe_2$|$SnSe_2$	27.87	21.50	24.40
PbS_2|$PbSe_2$	27.35	28.34	26.27
PbS_2|YS_2	17.76	28.34	2.51
$PbSe_2$|$SnTe_2$	26.88	26.27	27.37
$PbSe_2$|$ZrTe_2$	18.39	26.27	12.09
PtS_2|ZrS_2	13.06	30.95	2.04
ScS_2|SnS_2	18.11	9.43	26.56
ScS_2|$TiTe_2$	19.93	9.43	19.84
$ScTe_2$|$SnSe_2$	20.59	15.09	20.36
SnS_2|$SnSe_2$	21.39	26.56	20.36
SnS_2|$TiSe_2$	18.55	26.56	15.65
SnS_2|$TiTe_2$	23.55	26.56	19.84
SnS_2|ZrS_2	12.77	26.56	2.04
SnS_2|$ZrSe_2$	19.28	26.56	4.73
$SnSe_2$|$TiTe_2$	19.15	24.40	19.84
$SnSe_2$|$ZrTe_2$	17.15	24.40	12.09
VS_2|WS_2	19.12	21.50	15.97
ZrS_2|$ZrSe_2$	3.33	2.04	4.73

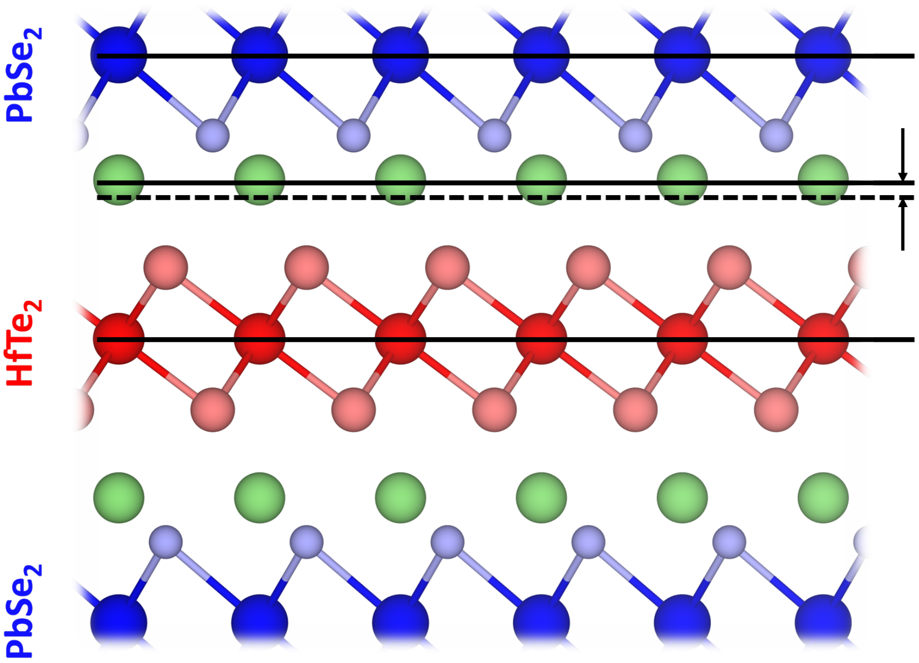

Figure S10: Asymmetry of intercalant position in superlattices that display values of E_{IS} greater than their component TMDCs, using the lithium-intercalated $HfTe_2|PbSe_2$ as an example. Solid lines indicate the planes of atoms, dashed line indicates the mid-point within the vdW space.

Energetics

We here show and discuss the intercalation energetics for the superlattice structures. The average voltages (over the intercalant concentration range considered) and E_{IS} values (at intercalant concentrations of $Li_2MX_2M'X'_2$ and $Mg_2MX_2M'X'_2$) for the superlattices and the relevant components are presented in Table S13 and Table S14 for lithium intercalation, and in Table S15 and Table S16 for magnesium intercalation. In each of these, the component voltages and E_{IS} values are also presented for easy comparison with the superlattice value. The components in a given pairing have been ordered alphabetically, and then each pairing listed in the tables alphabetically.

We find that, for the further pairings considered, the average values of voltage and E_{IS} of the component materials provide bounds for the values exhibited by the superlattice, and the average of these values provides a good estimate. We do highlight some exceptions to

this general rule, however.

For example, the values of E_{IS} for lithium-intercalated $HfTe_2|PbSe_2$, $HfTe_2|SnSe_2$, and $PbSe_2|ZrTe_2$, and magnesium-intercalated $CuS_2|RhS_2$, $SnS_2|TiTe_2$, and $SnSe_2|ZrTe_2$ exceed the values of the component materials. However, closer study of the geometry of these superlattices reveals a difference from their component TMDCs and the other superlattices: For most superlattices and component TMDCs, the intercalant species occupies a space in the vdW gap that close to half way between each of the neighbouring TMDC layers. However, for the exceptions listed above, the intercalant species is instead significantly closer to one of the component layers. For individual TMDCs this bias is not possible due to each layer being equivalent, and though there are some small deviations from the midpoint in other superlattices, these deviations are relatively small compared to the six exception highlight above. This is depicted in Figure S10, where the position of the lithium ions within the vdW spacing of $HfTe_2|PbSe_2$ (indicated with a solid line) lies away from the mid-point (indicated with a dashed line). This asymmetry is seen for each of the intercalant concentrations, and not just the $Li_2MX_2|M'X'_2$ and $Mg_2MX_2|M'X'_2$, though it becomes slightly more pronounced with higher intercalant concentrations.

Table S13: Average voltage and E_{IS}^{Li} values (corresponding to $Li_2MX_2M'X'_2$) for considered superlattices. The values of the component TMDCs[12] are also presented for easy comparison with the superlattice value.

Superlattice A|B	Superlattice Voltage (V)	Component A Voltage (V)	Component B Voltage (V)	E_{IS}^{Li} Superlattice (eV)	E_{IS}^{Li} A (eV)	E_{IS}^{Li} B (eV)
$CuS_2\|OsS_2$	2.41	2.23	2.57	0.29	0.04	0.62
$CuS_2\|RhS_2$	2.42	2.23	2.50	0.50	0.04	0.88
$GeS_2\|MoSe_2$	2.11	2.06	2.04	0.37	0.07	0.53
$GeS_2\|SnS_2$	1.98	2.06	1.80	0.03	0.07	-0.09
$GeS_2\|SnSe_2$	2.04	2.06	1.85	0.15	0.07	0.13
$GeS_2\|TiSe_2$	2.06	2.06	1.96	0.62	0.07	1.05
$GeSe_2\|HfS_2$	1.88	2.08	1.73	0.58	0.22	1.02
$GeSe_2\|NiSe_2$	1.87	2.08	1.69	0.04	0.22	-0.08
$GeSe_2\|SnSe_2$	2.01	2.08	1.85	0.21	0.22	0.13
$GeSe_2\|TaTe_2$	1.76	2.08	1.41	0.17	0.22	0.05
$GeSe_2\|TiSe_2$	2.03	2.08	1.96	0.64	0.22	1.05
$GeSe_2\|TiTe_2$	1.87	2.08	1.52	0.48	0.22	0.55
$HfS_2\|PdS_2$	1.92	1.73	1.94	0.60	1.02	-0.00
$HfS_2\|PtS_2$	1.60	1.73	1.43	0.43	1.02	-0.49
$HfS_2\|SnS_2$	1.75	1.73	1.80	0.45	1.02	-0.09
$HfS_2\|SnSe_2$	1.68	1.73	1.85	0.41	1.02	0.13
$HfS_2\|ZrS_2$	1.88	1.73	2.03	1.21	1.02	1.38
$HfTe_2\|PbSe_2$	2.17	1.25	2.22	0.90	0.34	0.39
$HfTe_2\|SnSe_2$	1.67	1.25	1.85	0.40	0.34	0.13
$MoS_2\|SnS_2$	2.26	2.47	1.80	0.54	1.05	-0.09
$MoS_2\|VS_2$	2.41	2.47	2.33	1.07	1.05	1.04
$MoS_2\|WS_2$	2.39	2.47	2.28	0.92	1.05	0.74
$MoS_2\|WSe_2$	1.86	2.47	1.90	0.22	1.05	0.24
$MoTe_2\|OsS_2$	1.71	1.60	2.57	-0.11	0.10	0.62
$NbS_2\|TaS_2$	2.12	2.23	2.01	0.99	1.14	0.84

Table S14: Average voltage and E_{IS}^{Li} values (corresponding to $Li_2MX_2M'X'_2$) for considered superlattices. The values of the component TMDCs[12] are also presented for easy comparison with the superlattice value.

Superlattice A|B	Superlattice Voltage (V)	Component A Voltage (V)	Component B Voltage (V)	E_{IS}^{Li} Superlattice (eV)	E_{IS}^{Li} A (eV)	E_{IS}^{Li} B (eV)
NiS_2|SnS_2	2.04	2.10	1.80	0.11	0.23	-0.09
NiS_2|TiS_2	2.26	2.10	2.33	0.91	0.23	1.51
$NiTe_2$|$PbSe_2$	1.82	1.18	2.22	0.24	-0.31	0.39
$NiTe_2$|$SnSe_2$	1.55	1.18	1.85	-0.04	-0.31	0.13
OsS_2|RhS_2	2.40	2.57	2.50	0.57	0.62	0.88
$OsTe_2$|$SnSe_2$	1.68	1.64	1.85	-0.06	-0.08	0.13
PbS_2|$PbSe_2$	2.41	2.62	2.22	0.46	0.57	0.39
PbS_2|YS_2	3.08	2.62	3.85	1.86	0.57	3.58
$PbSe_2$|$SnTe_2$	1.92	2.22	1.66	0.29	0.39	0.19
$PbSe_2$|$ZrTe_2$	2.23	2.22	1.51	1.01	0.39	0.73
PdS_2|PtS_2	1.73	1.94	1.43	-0.19	0.00	-0.49
PtS_2|ZrS_2	1.75	1.43	2.03	0.47	-0.49	1.38
ScS_2|SnS_2	2.69	3.66	1.80	1.52	3.22	-0.09
ScS_2|$TiTe_2$	2.28	3.66	1.52	1.55	3.22	0.55
$ScTe_2$|$SnSe_2$	2.11	2.40	1.85	0.98	1.89	0.13
SnS_2|$SnSe_2$	1.83	1.80	1.85	0.01	-0.09	0.13
SnS_2|$TiSe_2$	1.92	1.80	1.96	0.51	-0.09	1.05
SnS_2|$TiTe_2$	1.74	1.80	1.52	0.34	-0.09	0.55
SnS_2|ZrS_2	1.89	1.80	2.03	0.61	-0.09	1.38
SnS_2|$ZrSe_2$	1.87	1.80	1.81	0.58	-0.09	1.09
$SnSe_2$|$TiTe_2$	1.76	1.85	1.52	0.43	0.13	0.55
$SnSe_2$|$ZrSe_2$	1.83	1.85	1.81	0.60	0.13	1.09
$SnSe_2$|$ZrTe_2$	1.77	1.85	1.51	0.55	0.13	0.73
VS_2|WS_2	2.31	2.33	2.28	0.91	1.04	0.74
ZrS_2|$ZrSe_2$	1.90	2.03	1.81	1.20	1.38	1.09

Table S15: Average voltage and E_{IS}^{Mg} values (corresponding to $Mg_2MX_2M'X'_2$) for considered superlattices. The values of the component TMDCs[12] are also presented for easy comparison with the superlattice value.

Superlattice A|B	Superlattice Voltage (V)	Component A Voltage (V)	Component B Voltage (V)	E_{IS}^{Mg} Superlattice (eV)	E_{IS}^{Mg} A (eV)	E_{IS}^{Mg} B (eV)
CuS_2|OsS_2	1.49	1.54	1.10	-0.22	-0.05	-1.41
CuS_2|RhS_2	1.53	1.54	1.09	0.51	-0.05	-0.41
GeS_2|$MoSe_2$	0.85	1.35	0.35	-1.26	-0.02	-2.45
GeS_2|SnS_2	1.32	1.35	1.22	0.06	-0.02	0.01
GeS_2|$SnSe_2$	1.33	1.35	1.18	0.23	-0.02	0.42
GeS_2|$TiSe_2$	0.99	1.35	0.63	0.23	-0.02	0.52
$GeSe_2$|HfS_2	0.80	1.34	0.43	0.09	0.39	0.44
$GeSe_2$|$NiSe_2$	1.11	1.34	0.89	-0.19	0.39	-0.73
$GeSe_2$|$SnSe_2$	1.29	1.34	1.18	0.45	0.39	0.42
$GeSe_2$|$TaTe_2$	0.60	1.34	-0.06	-1.29	0.39	-2.79
$GeSe_2$|$TiSe_2$	0.99	1.34	0.63	0.49	0.39	0.52
$GeSe_2$|$TiTe_2$	0.84	1.34	0.37	0.17	0.39	0.01
HfS_2|PdS_2	0.92	0.43	1.16	0.39	0.44	-0.55
HfS_2|PtS_2	0.55	0.43	0.64	-0.76	0.44	-2.02
HfS_2|SnS_2	0.87	0.43	1.22	0.39	0.44	0.01
HfS_2|$SnSe_2$	0.80	0.43	1.18	0.36	0.44	0.42
HfS_2|ZrS_2	0.57	0.43	0.70	0.96	0.44	1.45
$HfTe_2$|$PbSe_2$	1.03	0.10	1.64	0.98	-0.58	1.57
$HfTe_2$|$SnSe_2$	0.71	0.10	1.18	0.19	-0.58	0.42
MoS_2|SnS_2	1.05	0.65	1.22	-0.64	-1.50	0.01
MoS_2|VS_2	0.77	0.65	0.87	-0.77	-1.50	-0.13
MoS_2|WS_2	0.46	0.65	0.28	-2.35	-1.50	-3.17
MoS_2|WSe_2	0.34	0.65	0.05	-2.80	-1.50	-3.97
$MoTe_2$|OsS_2	0.53	0.05	1.10	-2.27	-2.94	-1.41
NbS_2|TaS_2	0.59	0.77	0.41	-0.57	0.17	-1.32

Table S16: Average voltage and E_{IS}^{Mg} values (corresponding to $Mg_2MX_2M'X'_2$) for considered superlattices. The values of the component TMDCs[12] are also presented for easy comparison with the superlattice value.

Superlattice A|B	Superlattice Voltage (V)	Component A Voltage (V)	Component B Voltage (V)	E_{IS}^{Mg} Superlattice (eV)	E_{IS}^{Mg} A (eV)	E_{IS}^{Mg} B (eV)
NiS_2|SnS_2	1.28	1.22	1.22	-0.09	-0.26	0.01
NiS_2|TiS_2	1.10	1.22	0.86	0.72	-0.26	1.24
$NiTe_2$|$PbSe_2$	0.97	0.45	1.64	0.17	-0.86	1.57
$NiTe_2$|$SnSe_2$	0.79	0.45	1.18	-0.31	-0.86	0.42
OsS_2|RhS_2	1.07	1.10	1.09	-1.02	-1.41	-0.41
$OsTe_2$|$SnSe_2$	0.92	0.76	1.18	-0.40	-0.82	0.42
PbS_2|$PbSe_2$	1.80	1.98	1.64	1.60	1.75	1.57
PbS_2|YS_2	1.77	1.98	1.45	2.95	1.75	3.76
$PbSe_2$|$SnTe_2$	1.23	1.64	0.97	0.94	1.57	0.77
$PbSe_2$|$ZrTe_2$	1.08	1.64	0.30	1.25	1.57	0.33
PdS_2|PtS_2	0.94	1.14	0.63	-1.16	-0.55	-2.02
PtS_2|ZrS_2	0.67	0.64	0.70	-0.32	-2.02	1.45
ScS_2|SnS_2	1.38	1.52	1.22	1.92	3.68	0.01
ScS_2|$TiTe_2$	0.72	1.52	0.37	1.17	3.68	0.01
$ScTe_2$|$SnSe_2$	1.01	0.80	1.18	1.39	2.22	0.42
SnS_2|$SnSe_2$	1.20	1.22	1.18	0.17	0.01	0.42
SnS_2|$TiSe_2$	0.99	1.22	0.63	0.47	0.01	0.52
SnS_2|$TiTe_2$	0.86	1.22	0.37	0.24	0.01	0.01
SnS_2|ZrS_2	0.96	1.22	0.70	0.71	0.01	1.45
SnS_2|$ZrSe_2$	0.94	1.22	0.52	0.67	0.01	0.82
$SnSe_2$|$TiTe_2$	0.80	1.18	0.37	0.28	0.42	0.01
$SnSe_2$|$ZrSe_2$	0.83	1.18	0.52	0.52	0.42	0.82
$SnSe_2$|$ZrTe_2$	0.76	1.18	0.30	0.47	0.42	0.33
VS_2|WS_2	0.53	0.87	0.28	-1.82	-0.13	-3.17
ZrS_2|$ZrSe_2$	0.60	0.70	0.52	1.06	1.45	0.82

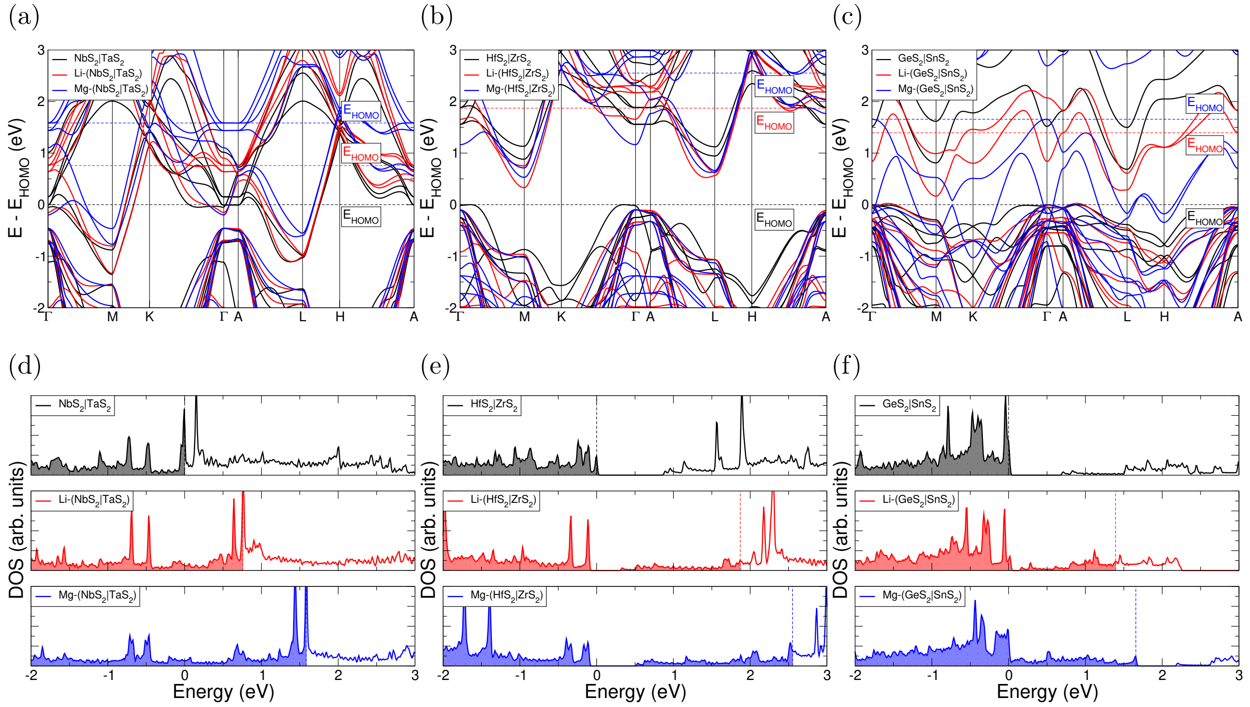

Figure S11: Electronic band structures and density of states (DOS) for pristine and intercalated superlattice structures. $NbS_2|TaS_2$ data is presented in S11a and S11d, $HfS_2|ZrS_2$ in S11b and S11e, and $GeS_2|SnS_2$ in S11c and S11f. Pristine data is presented in black, data for lithium-intercalated structures in red, and data for magnesium-intercalated structures in blue. Each has been aligned with high energy occupied states of the pristine superlattice material. The energy of the highest occupied state (E_{HOMO}) is indicated with dashed lines.

Electronic Structure

In the main article, we presented the electronic density of states (DOS) for selected superlattices to show the evolution of the highest occupied molecular orbital (HOMO) level with intercalation. Here, we also present the electronic structures of the superlattice structures in their bulk, lithium-intercalated, and magnesium-intercalated forms, corresponding to each of the DOS. In Figure S11 we present the electronic band structures and corresponding DOS for $NbS_2|TaS_2$ (S11a and S11d), $HfS_2|ZrS_2$ (S11b and S11e), and $GeS_2|SnS_2$ (S11c and S11f). These show the electronic structure for the unintercalated superlattices, along with the $2a = b = 2$ limit of lithium and magnesium intercalation. As was discussed in the 'Calculation Details' section, the presence of intercalants and local charge transfer lead to

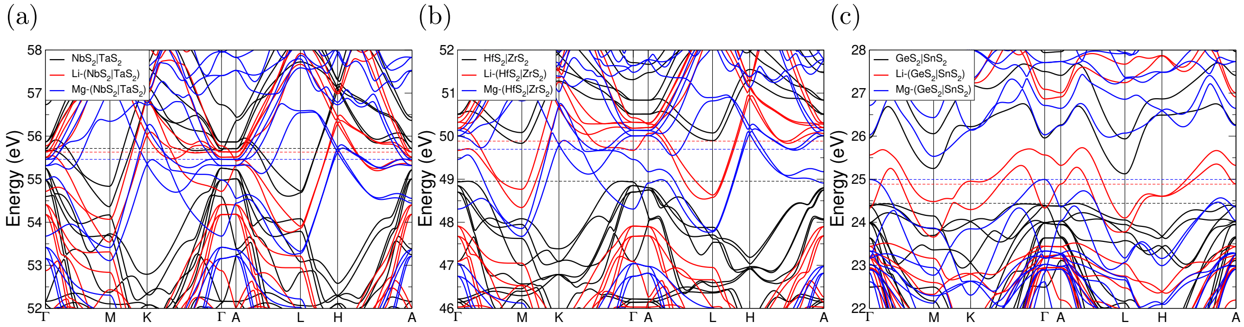

Figure S12: Electronic band structures with alternative alignment with respect to the lowest energy core states. The lowest energy states that have been aligned are set at 0 eV. $NbS_2|TaS_2$ data is presented in S12a, $HfS_2|ZrS_2$ in S12b, and $GeS_2|SnS_2$ in S12c. Pristine data is presented in black, data for lithium-intercalated structures in red, and data for magnesium-intercalated structures in blue. The energy of the highest occupied state is indicated with dashed lines.

local electric fields and so alignment of these band structures has been achieved through qualitatively aligned to the high-energy occupied states of the unintercalated superlattice at Γ, allowing us to comment on the relative position of the highest occupied molecular orbital (HOMO) level.

With the electronic band structures corresponding to the DOS presented in the main article, we now see that, whilst some bands experience static shifts or slight modification, the electronic structure of the host material changes relatively little when intercalated. Decomposition of the DOS into the orbital contributions shows that there are no states corresponding to the intercalant within the energy range presented. Hence, the addition of the electrons from the intercalant species to the host TMDC structure leads to a progressive upwards shift of the HOMO level, arising from electrons being donated from the intercalant to the states of the host TMDC structure.

Elastic Properties

The elastic matrices for each of the nine superlattices highlighted here have been determined. From these, several elastic properties can be calculated.

Elastic Tensors

Here we present the elements of the elastic matrix for each of the superlattices presented in the main article. We find each to possess a trigonal symmetry, and so the only unique non-zero elements are c_{11}, c_{12}, c_{13}, c_{33}, c_{44}, c_{14}, and c_{66}. These values are presented in Table S17.

We find that most of the pristine, lithium-intercalated, and magnesium-intercalated structures are elastically stable, by assessing the Born stability criteria outlined elsewhere.[19] However, we find that magnesium-intercalated $HfS_2|PdS_2$ is elastically unstable, breaking the same conditions that are broken by magnesium-intercalated PdS_2. Further, magnesium-intercalated $MoS_2|SnS_2$ is also found to be elastically unstable (breaking the requirements of $c_{11} > |c_{12}|$ and $c_{14}^2 < \frac{1}{2}c_{44}(c_{11} - c_{12}) = c_{44}c_{66}$) despite neither of its components being elastically unstable. This could be due to the larger strain on each of these systems ($> 5\%$) compared to the other superlattices. With the introduction of an intercalant, we find the space group of several superlattices is changed from 164 to 156.

Table S17: Elastic matrix elements for each of the pristine and intercalated superlattices presented in the main article. Materials marked with an asterisk (*) are elastically unstable.

A|B	Int.	Space Group	c_{11} (GPa)	c_{12} (GPa)	c_{13} (GPa)	c_{33} (GPa)	c_{44} (GPa)	c_{14} (GPa)	c_{66} (GPa)
SnS_2|$SnSe_2$	-	164	152.89	62.72	80.35	119.75	30.47	-18.10	45.08
	Li	156	83.37	40.89	19.70	77.08	15.95	-7.54	21.24
	Mg	156	96.91	39.75	39.17	125.09	36.23	-12.71	28.58
NiS_2|TiS_2	-	164	165.24	43.47	13.50	40.72	15.90	6.86	60.88
	Li	156	124.30	20.18	23.04	112.21	31.27	-3.59	52.06
	Mg	156	157.61	65.46	62.44	179.28	49.61	-0.55	46.08
HfS_2|PdS_2	-	164	148.85	36.23	12.41	29.92	12.03	5.48	56.31
	Li	156	136.92	47.48	30.99	102.28	20.98	1.59	44.72
	Mg*	156	114.50	67.12	52.48	140.55	-2.97	-16.24	23.69
ZrS_2|$ZrSe_2$	-	164	120.91	24.50	7.54	34.58	9.30	2.64	48.20
	Li	156	135.85	37.92	28.24	111.03	37.96	6.48	48.97
	Mg	156	151.39	51.24	74.39	162.33	67.45	6.86	50.07
NbS_2|TaS_2	-	164	155.31	34.32	8.51	44.50	9.81	4.27	60.50
	Li	156	152.61	51.06	58.64	129.16	53.36	10.22	50.77
	Mg	156	182.65	65.78	101.48	203.05	84.51	6.52	58.43
GeS_2|SnS_2	-	164	127.17	37.63	10.10	27.90	8.42	-0.64	44.77
	Li	164	79.12	57.07	21.25	89.80	19.81	5.33	11.03
	Mg	164	114.01	43.64	41.31	143.95	42.71	12.74	35.18
$SnSe_2$|$ZrTe_2$	-	164	85.371	23.26	10.28	25.63	7.90	1.19	31.06
	Li	164	75.83	31.28	19.29	66.46	24.42	1.97	22.27
	Mg	164	82.63	31.95	36.55	56.64	4.26	4.31	25.34
HfS_2|ZrS_2	-	164	140.94	27.52	7.82	34.16	9.02	-2.66	56.71
	Li	156	155.44	47.53	28.09	124.63	39.92	-7.29	53.95
	Mg	156	177.55	59.78	82.87	185.80	77.99	-8.50	58.88
MoS_2|SnS_2	-	164	146.62	33.82	11.02	31.64	12.49	4.83	56.40
	Li	156	116.28	44.45	36.15	111.85	34.50	7.20	35.91
	Mg*	156	66.93	93.37	64.66	111.96	0.23	6.21	-13.22

Elastic Moduli

Here, we present the polycrystalline bulk (B), shear (G), and Young's (Y) moduli for each of the superlattice structures presented in the main article. We also include the Poisson (ν) and Pugh (R) elastic ratios which are commonly used to describe the ductility of a material. A ductile material typically has a Poisson ratio greater than 0.26, and Pugh ratios greater than 1.75. Each of these quantities has been calculated using the elastic matrices presented above and the Voigt-Reuss-Hill average scheme. With the exception of $SnS_2|SnSe_2$, we find that the bulk modulus of the superlattice is increased with the introduction of an intercalant, with a larger increase seen for lithium than for magnesium intercalation. This indicates the increased interaction between TMDC layers, with the ionic intercalant occupying the vdW spacing.

Table S18: Elastic properties for each of the pristine and intercalated superlattices highlighted in this Chapter. These have been calculated using the Voigt-Reuss-Hill average scheme. Materials marked with an asterisk (*) are elastically unstable.

A|B	Intercalant	B (GPa)	G (GPa)	Y (GPa)	ν	R
SnS_2|$SnSe_2$	-	96.73	30.53	82.87	0.36	3.17
	Li	44.51	19.26	50.49	0.31	2.31
	Mg	61.31	31.11	79.82	0.28	1.97
NiS_2|TiS_2	-	45.67	31.27	76.38	0.22	1.46
	Li	54.78	41.29	99.00	0.20	1.33
	Mg	97.12	49.21	126.31	0.28	1.97
HfS_2|PdS_2	-	38.37	26.24	64.10	0.22	1.46
	Li	65.02	32.81	84.26	0.28	1.98
	Mg*	79.24	-16.55	-53.38	0.61	-4.79
ZrS_2|$ZrSe_2$	-	33.08	22.67	55.36	0.22	1.46
	Li	63.04	43.43	105.95	0.22	1.45
	Mg	95.56	53.11	134.42	0.27	1.80
NbS_2|TaS_2	-	42.39	27.02	66.86	0.24	1.57
	Li	85.55	48.11	121.54	0.26	1.78
	Mg	121.46	62.82	160.74	0.28	1.93
GeS_2|SnS_2	-	34.30	20.93	52.19	0.25	1.64
	Li	49.38	17.14	46.08	0.34	2.88
	Mg	69.08	38.39	97.17	0.27	1.80
$SnSe_2$|$ZrTe_2$	-	26.61	16.13	40.27	0.25	1.65
	Li	39.45	23.94	59.73	0.25	1.65
	Mg	47.38	10.67	29.76	0.40	4.44
HfS_2|ZrS_2	-	36.05	24.68	60.29	0.22	1.46
	Li	70.61	47.71	116.82	0.22	1.48
	Mg	109.82	62.36	157.31	0.26	1.76
MoS_2|SnS_2	-	37.94	26.89	65.25	0.21	1.41
	Li	64.14	35.48	89.86	0.27	1.81
	Mg*	76.57	3.21	9.48	0.48	23.89

(a)

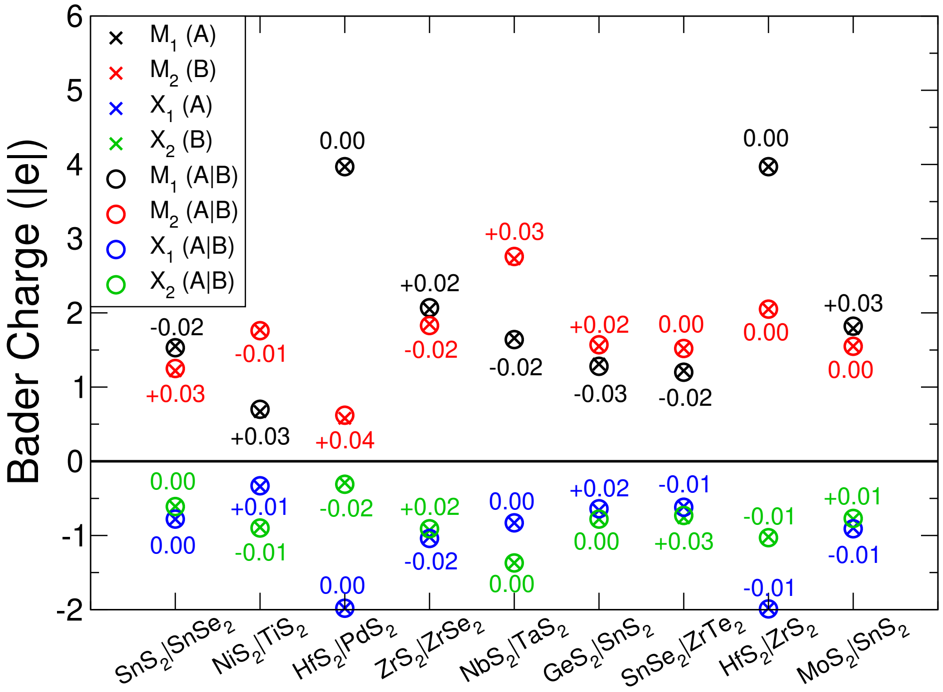

Figure S13: Bader charges for the different metal and chalcogen species in the unintercalated pristine superlattices and the relevant component TMDCs. The included numbers indicate the difference in charge between the superlattice and individual TMDC components, $Q^{SL} - Q^{TMDC}$.

Charge Analysis

The first consideration is of charge transfer changes during the construction of superlattices and during intercalation. Upon construction of a superlattice, and more importantly upon intercalation, there can be large charge transfers between the constituent atoms. The magnitude of this charge transfer and where the charge is transferred to/from plays an important role in determining how much energy is involved with forming a superlattice or intercalating a layered material. Numerical values are presented in Table S19 - Table S27.

Superlattice Construction

Upon construction of a superlattice (without the inclusion of any intercalant), we would expect minimal charge transfer between the component layers due to the presence of the

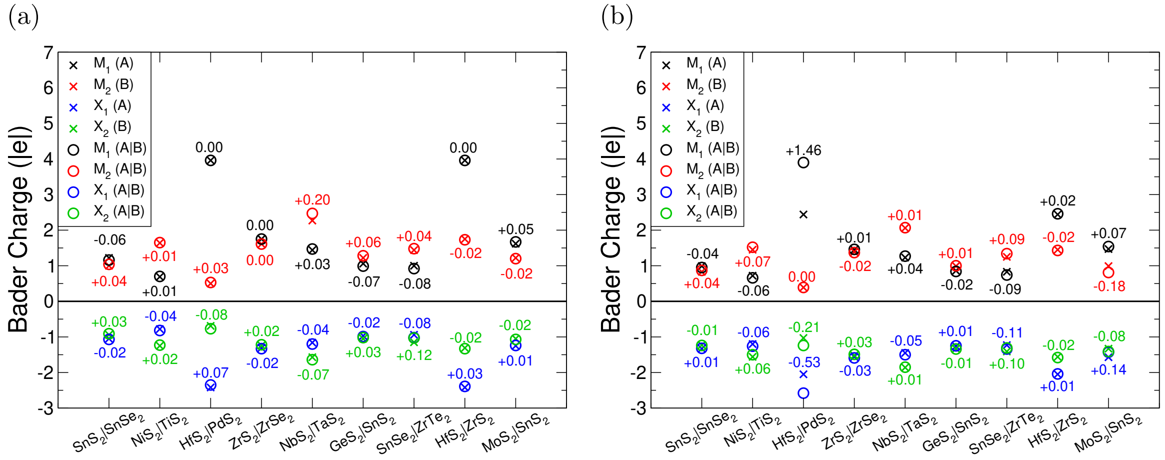

Figure S14: Bader charges for the different metal and chalcogen species in superlattices and the relevant component TMDCs, when fully intercalated (i.e. one intercalant per metal of the host structure) with lithium (S14a) and magnesium (S14b). The included numbers indicate the difference in charge between the superlattice and individual TMDC components, $Q^{SL} - Q^{TMDC}$.

vdW gap. In Figure S13 we present the Bader charges of the species in the highlighted superlattices, along with the Bader charges of the species in the individual components. We find that the charges on both the metal and chalcogen species are largely preserved compared to their charges in the individual MX_2 components. For the pristine systems, Bader charges are shown in Figure S13a, where the charges of the metal and chalcogen species are seen to be effectively unchanged between the individual pristine TMDCs and the superlattices. We highlight this with the numbers presented on Figure S13a, which give the difference in charge on an ionic species in the superlattice and in the component TMDC, i.e. $Q^{SL} - Q^{TMDC}$. In fact, the largest difference between the component and superlattice is found to be 0.04 |e| in the $HfS_2|PdS_2$ system.

Intercalated Superlattices

We compare the Bader charges for the lithium-intercalated (Figure S14a) and magnesium-intercalated (Figure S14b) systems, when fully intercalated such that there is one intercalant per metal of the host structure . Surprisingly, whilst there is more charge transfer than is

seen with the pristine systems, this remains relatively small. We highlight this with the numbers presented on Figure S14a and Figure S14b, which give the difference in charge on an ionic species in the superlattice and in the component TMDC, i.e. $Q^{SL} - Q^{TMDC}$. It is now clear to see that the charge of most of the ionic species differ by less that 0.1 |e|. However, some systems, for example lithium-intercalated $NbS_2|TaS_2$ and magnesium-intercalated $HfS_2|PdS_2$, show significant charge transfer between the component layers. The intercalants themselves maintain almost constant charges, as has been shown for intercalation into the individual TMDCs.[12] Across the different superlattices, the charge of lithium varies between $0.87 - 0.88$, and magnesium varies between $1.65 - 1.67$.

To supplement the results of the Bader charge analysis, we have also considered the differences in the charge density arising from intercalation: Whilst maintaining the positions of the constituent atoms, the electronic charge densities were obtained, and compared using $\Delta\rho = \rho_{\text{LiSL}} - [\rho_{\text{Li}} + \rho_{\text{SL}}]$. We present in Figure S15 the planar-averaged values of $\Delta\rho$ for the $SnS_2|SnSe_2$ superlattice intercalated with lithium (Figure S15a) and magnesium (Figure S15b). The results for the component SnS_2 and $SnSe_2$ structures have also been included.

For $SnS_2|SnSe_2$, the tin atoms (purple) are positioned at $c = 0.25$ and $c = 0.75$, the chalcogen (yellow sulfur, green selenium) atoms are positioned at c values ± 0.125 either side of these, and the intercalant species (orange) are positioned at $c = 0$ (periodic image at $c = 1$) and $c = 0.5$. For both lithium and magnesium, we see significant electron depletion from the intercalant regions (at $c = 0, 0.5, 1$) as these species donate electrons to the parent superlattice structure. This charge is seen to accumulate in the bonding regions between chalcogen and intercalant. Due to this additional charge on the chalcogen species, the electrons used in the M-X bond are able to redistribute back to the host metal. This is seen with a depletion of electronic charge between the metal and chalcogen ($c = 0.15 - 0.20, c = 0.30 - 0.35, c = 0.65 - 0.70, c = 0.80 - 0.85$), and by a charge donation to the metal. We find that charge transfer to each of the layers in the superlattice closely matches the

charge transfer seen for the respective TMDC on its own. For example, with magnesium intercalation, the charge transfer from the intercalated magnesium to the SnS_2 layer of the superlattice (Figure S15b, purple line, $c = 0-0.5$) very closely resembles the profile of charge transfer seen for magnesium intercalation into SnS_2 (same figure, red line).

We continue our discussion of charge analysis in Figure S15c and Figure S15d, which depict 3D visualisations of this charge transfer for lithium and magnesium intercalation, respectively. The isosurfaces chosen are the chosen by the ratio of intercalant Bader charges ($\frac{q_{Mg}}{q_{Li}} = \frac{1.65}{0.88}$). In Figure S15e, we further show a 2D slice through this charge difference along the (1 1 0) plane, passing through host metal atoms, chalcogen atoms, and the intercalated lithium. In each of these 2D and 3D visualisations, red isosurfaces show electron depletion and blue isosurfaces show electron accumulation. These offer further detail of the structure of the charge transfer, and show the similarity for both lithium and magnesium intercalation.

We find a very similar results to those presented in the above discussion for the other superlattices, examples of which have been presented in Figure S16.

As the charge transfer upon construction of the superlattice remains small, and the charge transfer that follows the inclusion of an intercalant mirrors the transfer that arises in each of the constituent TMDC layers, it is therefore clear as to why the superlattice energetics (i.e. the intercalation voltage and the stability metric of E_{IS}) take on intermediate values to those of the component TMDCs.

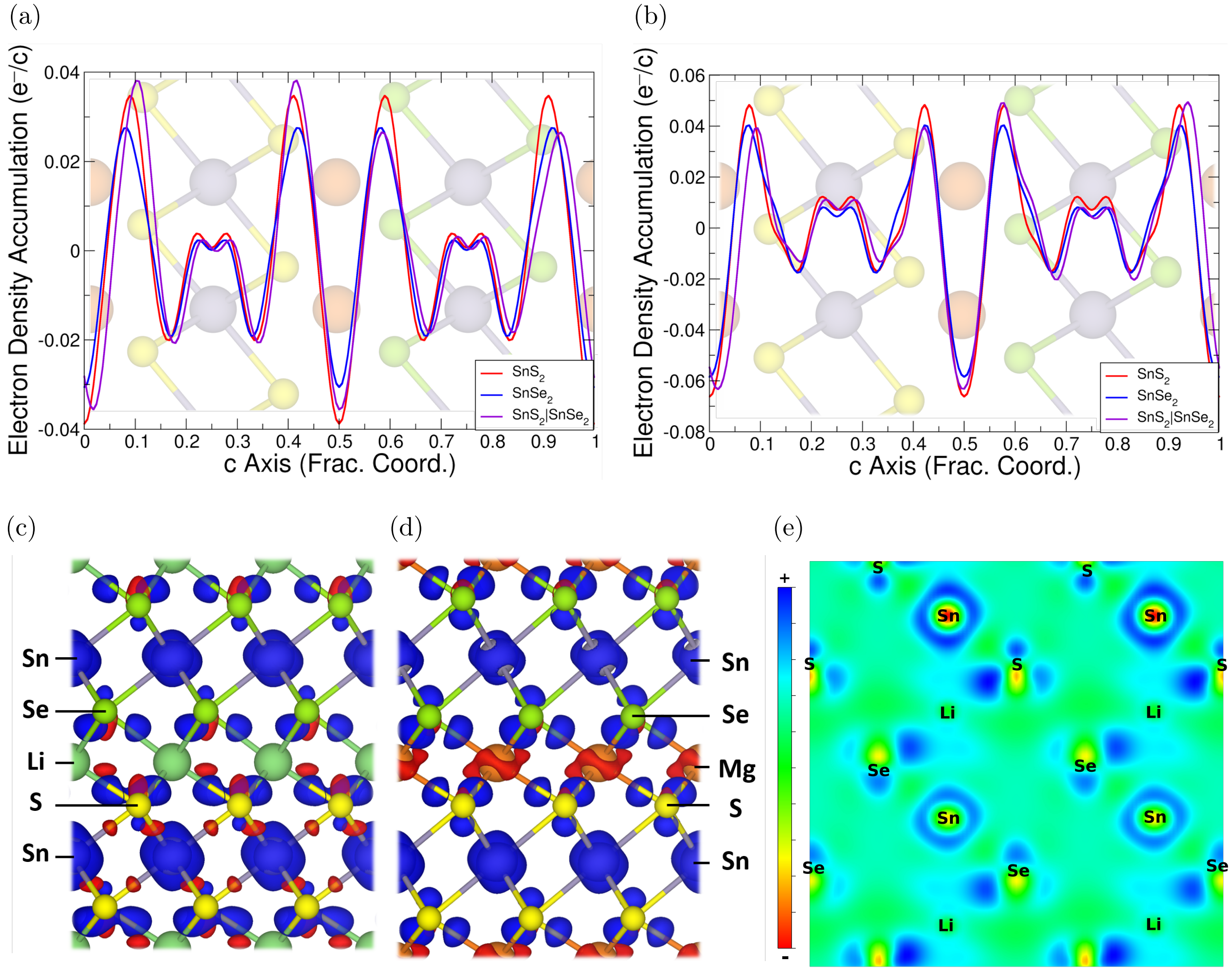

Figure S15: The planar-average of $\Delta\rho = \rho_{\mathrm{LiSL}} - [\rho_{\mathrm{Li}} + \rho_{\mathrm{SL}}]$ for the $SnS_2|SnSe_2$ superlattice (and the component materials) intercalated with lithium (S15a) and magnesium (S15b). Positive values correspond to regions of electron accumulation, and negative values correspond to regions of electron depletion. The corresponding structure is overlayed on these plots, with purple tin atoms, yellow sulfur, green selenium, and orange intercalant. The 3D visualisation of this charge transfer in $SnS_2|SnSe_2$ is shown in S15c and S15d for lithium (isosurface 2.5 me$^-$/Å^3) and magnesium (isosurface 4.7 me$^-$/Å^3) intercalation, respectively. S15e shows a 2D slice through the (1 1 0) plane of the Li-($SnS_2|SnSe_2$) charge-difference distribution. Red isosurfaces show electron depletion and blue isosurfaces show electron accumulation.

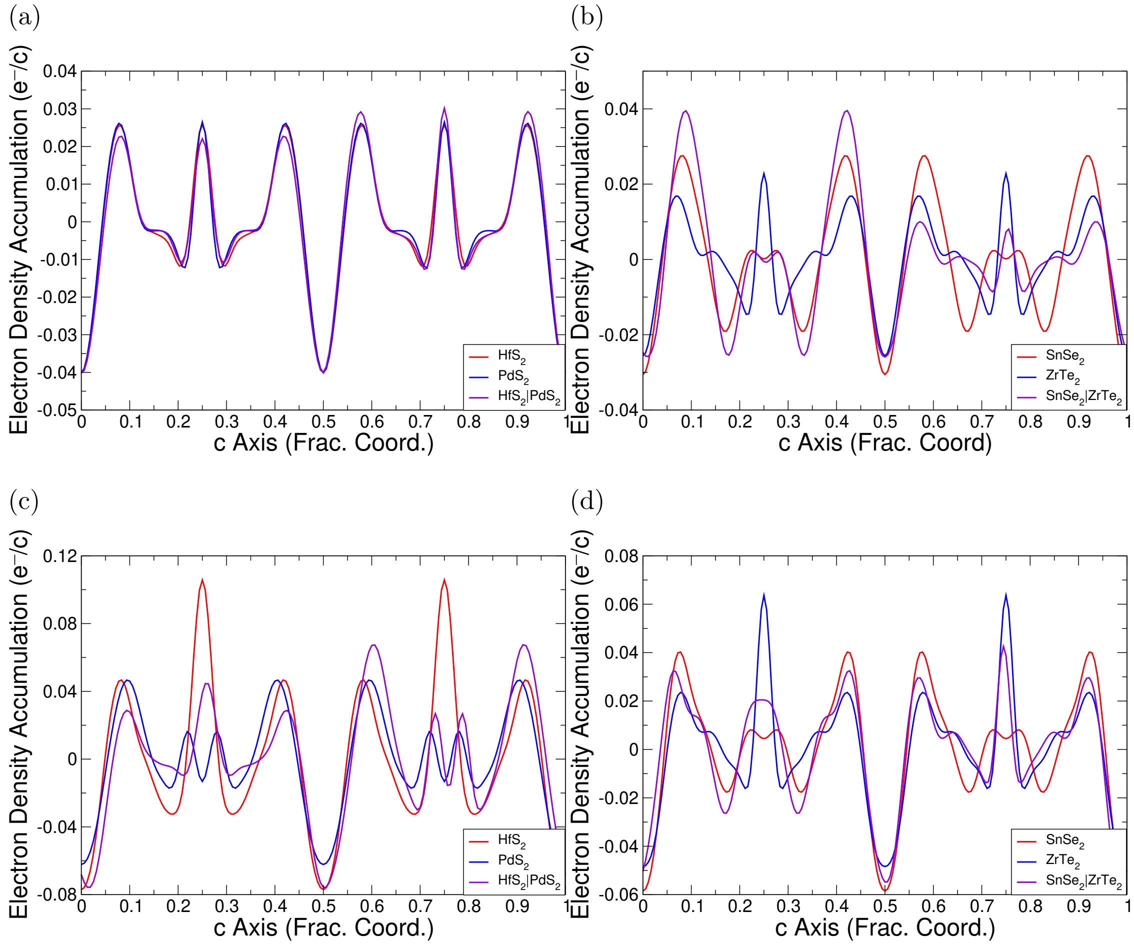

Figure S16: The planar-average charge difference plots for lithium-intercalated $HfS_2|PdS_2$ (S16a) and $SnSe_2|ZrTe_2$ (S16b). Similar plots for magnesium-intercalated $HfS_2|PdS_2$ (S16c) and $SnSe_2|ZrTe_2$ (S16d) have also been included. Positive values correspond to regions of electron accumulation, and negative values correspond to regions of electron depletion.

Charge Tables

We present in Table S19 - Table S27 the numerical Bader charge values for each of the superlattice structures and their component TMDCs, each in their pristine and intercalated forms.

Table S19: Bader charge values for SnS_2, $SnSe_2$, and their superlattice.

Species	Charge of species in bulk SnS_2 (|e|)	Charge of species in bulk $SnSe_2$ (|e|)	Charge of species in bulk superlattice (|e|)
Sn_1	1.55	-	1.53
S	-0.78	-	-0.78
Sn_2	-	1.22	1.25
Se	-	-0.61	-0.61
Species	Charge of species in intercalated $LiSnS_2$ (|e|)	Charge of species in intercalated $LiSnSe_2$ (|e|)	Charge of species in intercalated superlattice (|e|)
Sn_1	1.22	-	1.16
S	-1.05	-	-1.07
Sn_2	-	1.00	1.04
Se	-	-0.94	-0.91
Li	0.88	0.87	0.88
Species	Charge of species in intercalated $MgSnS_2$ (|e|)	Charge of species in intercalated $MgSnSe_2$ (|e|)	Charge of species in intercalated superlattice (|e|)
Sn_1	0.99	-	0.95
S	-1.33	-	-1.32
Sn_2	-	0.83	0.87
Se	-	-1.23	-1.24
Mg	1.67	1.63	1.65

Table S20: Bader charge values for NiS_2, TiS_2, and their superlattice.

Species	Charge of species in bulk NiS_2 (|e|)	Charge of species in bulk TiS_2 (|e|)	Charge of species in bulk superlattice (|e|)
Ni	0.67	-	0.70
S_1	-0.34	-	-0.33
Ti	-	1.77	1.76
S_2	-	-0.89	-0.90
Species	Charge of species in intercalated $LiNiS_2$ (|e|)	Charge of species in intercalated $LiTiS_2$ (|e|)	Charge of species in intercalated superlattice (|e|)
Ni	0.69	-	0.70
S_1	-0.78	-	-0.82
Ti	-	1.64	1.65
S_2	-	-1.25	-1.23
Li	0.87	0.87	0.87
Species	Charge of species in intercalated $MgNiS_2$ (|e|)	Charge of species in intercalated $MgTiS_2$ (|e|)	Charge of species in intercalated superlattice (|e|)
Ni	0.72	-	0.66
S_1	-1.20	-	-1.26
Ti	-	1.45	1.52
S_2	-	-1.56	-1.50
Mg	1.68	1.68	1.68

Table S21: Bader charge values for HfS_2, PdS_2, and their superlattice.

Species	Charge of species in bulk HfS_2 (|e|)	Charge of species in bulk PdS_2 (|e|)	Charge of species in bulk superlattice (|e|)
Hf	3.97	-	3.97
S_1	-1.98	-	-1.98
Pd	-	0.58	0.62
S_2	-	-0.29	-0.31
Species	Charge of species in intercalated $LiHfS_2$ (|e|)	Charge of species in intercalated $LiPdS_2$ (|e|)	Charge of species in intercalated superlattice (|e|)
Hf	3.96	-	3.96
S_1	-2.42	-	-2.35
Pd	-	0.50	0.53
S_2	-	-0.69	-0.77
Li	0.87	0.87	0.87
Species	Charge of species in intercalated $MgHfS_2$ (|e|)	Charge of species in intercalated $MgPdS_2$ (|e|)	Charge of species in intercalated superlattice (|e|)
Hf	2.44	-	3.90
S_1	-2.05	-	-2.58
Pd	-	0.39	0.39
S_2	-	-1.03	-1.24
Mg	1.67	1.68	1.66

Table S22: Bader charge values for ZrS_2, $ZrSe_2$, and their superlattice.

Species	Charge of species in bulk ZrS_2 (|e|)	Charge of species in bulk $ZrSe_2$ (|e|)	Charge of species in bulk superlattice (|e|)
Zr_1	2.05	-	2.07
S	-1.02	-	-1.04
Zr_2	-	1.85	1.83
Se	-	-0.93	-0.91
Species	Charge of species in intercalated $LiZrS_2$ (|e|)	Charge of species in intercalated $LiZrSe_2$ (|e|)	Charge of species in intercalated superlattice (|e|)
Zr_1	1.75	-	1.75
S	-1.31	-	-1.33
Zr_2	-	1.61	1.61
Se	-	-1.24	-1.22
Li	0.87	0.87	0.87
Species	Charge of species in intercalated $MgZrS_2$ (|e|)	Charge of species in intercalated $MgZrSe_2$ (|e|)	Charge of species in intercalated superlattice (|e|)
Zr_1	1.45	-	1.46
S	-1.56	-	-1.59
Zr_2	-	1.39	1.37
Se	-	-1.52	-1.49
Mg	1.68	1.64	1.66

Table S23: Bader charge values for NbS_2, TaS_2, and their superlattice.

Species	Charge of species in bulk NbS_2 (|e|)	Charge of species in bulk TaS_2 (|e|)	Charge of species in bulk superlattice (|e|)
Nb	1.66	-	1.64
S_1	-0.83	-	-0.83
Ta	-	2.73	2.76
S_2	-	-1.37	-1.37
Species	Charge of species in intercalated $LiNbS_2$ (|e|)	Charge of species in intercalated $LiTaS_2$ (|e|)	Charge of species in intercalated superlattice (|e|)
Nb	1.44	-	1.47
S_1	-1.16	-	-1.20
Ta	-	2.27	2.47
S_2	-	-1.57	-1.64
Li	0.87	0.88	0.87
Species	Charge of species in intercalated $MgNbS_2$ (|e|)	Charge of species in intercalated $MgTaS_2$ (|e|)	Charge of species in intercalated superlattice (|e|)
Nb	1.23	-	1.27
S_1	-1.45	-	-1.50
Ta	-	2.06	2.07
S_2	-	-1.86	-1.85
Mg	1.68	1.67	1.67

Table S24: Bader charge values for GeS_2, SnS_2, and their superlattice.

Species	Charge of species in bulk GeS_2 (|e|)	Charge of species in bulk SnS_2 (|e|)	Charge of species in bulk superlattice (|e|)
Ge	1.31	-	1.28
S_1	-0.66	-	-0.64
Sn	-	1.55	1.57
S_2	-	-0.78	-0.78
Species	Charge of species in intercalated $LiGeS_2$ (|e|)	Charge of species in intercalated $LiSnS_2$ (|e|)	Charge of species in intercalated superlattice (|e|)
Ge	1.06	-	0.99
S_1	-0.97	-	-0.99
Sn	-	1.22	1.28
S_2	-	-1.05	-1.02
Li	0.88	0.88	0.88
Species	Charge of species in intercalated $MgGeS_2$ (|e|)	Charge of species in intercalated $MgSnS_2$ (|e|)	Charge of species in intercalated superlattice (|e|)
Ge	0.86	-	0.84
S_1	-1.26	-	-1.25
Sn	-	0.99	1.00
S_2	-	-1.33	-1.34
Mg	1.67	1.67	1.67

Table S25: Bader charge values for $SnSe_2$, $ZrTe_2$, and their superlattice.

Species	Charge of species in bulk $SnSe_2$ (|e|)	Charge of species in bulk $ZrTe_2$ (|e|)	Charge of species in bulk superlattice (|e|)
Sn	1.22	-	1.20
Se	-0.61	-	-0.62
Zr	-	1.52	1.52
Te	-	-0.76	-0.73
Species	Charge of species in intercalated $LiSnSe_2$ (|e|)	Charge of species in intercalated $LiZrTe_2$ (|e|)	Charge of species in intercalated superlattice (|e|)
Sn	1.00	-	0.92
Se	-0.94	-	-1.02
Zr	-	1.44	1.48
Te	-	-1.15	-1.03
Li	0.87	0.86	0.87
Species	Charge of species in intercalated $MgSnSe_2$ (|e|)	Charge of species in intercalated $MgZrTe_2$ (|e|)	Charge of species in intercalated superlattice (|e|)
Sn	0.83	-	0.74
Se	-1.23	-	-1.34
Zr	-	1.25	1.34
Te	-	-1.41	-1.31
Mg	1.63	1.59	1.60

Table S26: Bader charge values for HfS_2, ZrS_2, and their superlattice.

Species	Charge of species in bulk HfS_2 (|e|)	Charge of species in bulk ZrS_2 (|e|)	Charge of species in bulk superlattice (|e|)
Hf	3.97	-	3.97
S_1	-1.98	-	-1.99
Zr	-	2.05	2.05
S_2	-	-1.02	-1.03
Species	Charge of species in intercalated $LiHfS_2$ (|e|)	Charge of species in intercalated $LiZrS_2$ (|e|)	Charge of species in intercalated superlattice (|e|)
Hf	3.96	-	3.96
S_1	-2.42	-	-2.39
Zr	-	1.75	1.73
S_2	-	-1.31	-1.33
Li	0.87	0.87	0.87
Species	Charge of species in intercalated $LiHfS_2$ (|e|)	Charge of species in intercalated $LiZrS_2$ (|e|)	Charge of species in intercalated superlattice (|e|)
Hf	2.44	-	2.46
S_1	-2.05	-	-2.04
Zr	-	1.45	1.43
S_2	-	-1.56	-1.58
Mg	1.67	1.68	1.67

Table S27: Bader charge values for MoS_2, SnS_2, and their superlattice.

Species	Charge of species in bulk MoS_2 (|e|)	Charge of species in bulk SnS_2 (|e|)	Charge of species in bulk superlattice (|e|)
Mo	1.79	-	1.82
S_1	-0.90	-	-0.91
Sn	-	1.55	1.55
S_2	-	-0.78	-0.77
Species	Charge of species in intercalated $LiMoS_2$ (|e|)	Charge of species in intercalated $LiSnS_2$ (|e|)	Charge of species in intercalated superlattice (|e|)
Mo	1.62	-	1.67
S_1	-1.25	-	-1.24
Sn	-	1.22	1.20
S_2	-	-1.05	-1.07
Li	0.87	0.88	0.87
Species	Charge of species in intercalated $MgMoS_2$ (|e|)	Charge of species in intercalated $MgSnS_2$ (|e|)	Charge of species in intercalated superlattice (|e|)
Mo	1.47	-	1.54
S_1	-1.57	-	-1.43
Sn	-	0.99	0.81
S_2	-	-1.33	-1.41
Mg	1.67	1.67	1.67

References

(1) Kresse, G.; Hafner, J. Ab initio molecular dynamics for liquid metals. *Physical Review B* **1993**, *47*, 558–561.

(2) Kresse, G.; Hafner, J. Ab initio molecular-dynamics simulation of the liquid-metal–amorphous-semiconductor transition in germanium. *Physical Review B* **1994**, *49*, 14251–14269.

(3) Kresse, G.; Furthmüller, J. Efficiency of ab-initio total energy calculations for metals and semiconductors using a plane-wave basis set. *Computational Materials Science* **1996**, *6*, 15–50.

(4) Kresse, G.; Furthmüller, J. Efficient iterative schemes for ab initio total-energy calculations using a plane-wave basis set. *Physical Review B* **1996**, *54*, 11169–11186.

(5) Blöchl, P. E. Projector augmented-wave method. *Physical Review B* **1994**, *50*, 17953–17979.

(6) Perdew, J. P.; Burke, K.; Ernzerhof, M. Generalized Gradient Approximation Made Simple. *Physical Review Letters* **1996**, *77*, 3865–3868.

(7) Monkhorst, H. J.; Pack, J. D. Special points for Brillouin-zone integrations. *Physical Review B* **1976**, *13*, 5188–5192.

(8) Grimme, S.; Antony, J.; Ehrlich, S.; Krieg, H. A consistent and accurate ab initio parametrization of density functional dispersion correction (DFT-D) for the 94 elements H-Pu. *The Journal of Chemical Physics* **2010**, *132*, 154104.

(9) Taylor, N. T.; Price, C. J.; Petkov, A.; Romanis Carr, M. I.; Hale, J. C.; Hepplestone, S. P. The Potential of Overlayers on Tin-based Perovskites for Water Splitting. *Journal of Physical Chemistry Letters* **2020**, *11*, 4124–4130.

(10) Davies, F. H.; Price, C. J.; Taylor, N. T.; Davies, S. G.; Hepplestone, S. P. Band alignment of transition metal dichalcogenide heterostructures. *Physical Review B* **2021**, *103*, 1–10.

(11) Wei, S. H.; Zunger, A. Calculated natural band offsets of all II-VI and III-V semiconductors: Chemical trends and the role of cation d orbitals. *Applied Physics Letters* **1998**, *72*, 2011–2013.

(12) Price, C. J.; Baker, E. A. D.; Hepplestone, S. P. First principles study of layered transition metal dichalcogenides for use as electrodes in Li-ion and Mg-ion batteries. *Journal of Materials Chemistry A* **2023**, *11*, 12354–12372.

(13) Henkelman, G.; Uberuaga, B. P.; Jónsson, H. Climbing image nudged elastic band method for finding saddle points and minimum energy paths. *Journal of Chemical Physics* **2000**, *113*, 9901–9904.

(14) Fan, S.; Zou, X.; Du, H.; Gan, L.; Xu, C.; Lv, W.; He, Y. B.; Yang, Q. H.; Kang, F.; Li, J. Theoretical Investigation of the Intercalation Chemistry of Lithium/Sodium Ions in Transition Metal Dichalcogenides. *Journal of Physical Chemistry C* **2017**, *121*, 13599–13605.

(15) Van der Ven, A.; Bhattacharya, J.; Belak, A. A. Understanding Li Diffusion in Li-Intercalation Compounds. *Accounts of Chemical Research* **2013**, *46*, 1216–1225.

(16) Zhang, Q.; Li, R.; Zhang, M.; Zhang, B.; Gou, X. SnS2/reduced graphene oxide nanocomposites with superior lithium storage performance. *Electrochimica Acta* **2014**, *115*, 425–433.

(17) Gao, P.; Wang, L.; Zhang, Y. Y.; Huang, Y.; Liao, L.; Sutter, P.; Liu, K.; Yu, D.; Wang, E. G. High-Resolution Tracking Asymmetric Lithium Insertion and Extraction and Local Structure Ordering in SnS2. *Nano Letters* **2016**, *16*, 5582–5588.

(18) Lu, Y.; Li, J.; Zhao, Y.; Zhu, X. Lithium Clustering during the Lithiation/Delithiation Process in LiFePO 4 Olivine-Structured Materials. *ACS Omega* **2019**, *4*, 20612–20617.

(19) Mouhat, F.; Coudert, F.-X. Necessary and sufficient elastic stability conditions in various crystal systems. *Physical Review B* **2014**, *90*, 224104.
